# Stability and Reactivity of ${\left({\mathrm{TiO}}_{2}\right)}_{n}$, *n* = 1–10, Clusters and Their Interactions With ${\mathrm{CO}}_{2}$

**DOI:** 10.1002/jcc.70232

**Published:** 2025-09-20

**Authors:** Letícia Carolaine Silva Faria, Letícia Marques de Souza Vetrano de Queiroz, Murielly Fernanda Ribeiro Bihain, Douglas Henrique Pereira, Leonardo Tsuyoshi Ueno, Francisco Bolivar Correto Machado, Luiz Fernando de Araujo Ferrão

**Affiliations:** ^1^ Aeronautics Institute of Technology (ITA) São José dos Campos SP Brazil; ^2^ Department of Chemistry Federal University of Tocantins (UFT) Gurupi TO Brazil

## Abstract

Small titanium dioxide clusters ${\left({\mathrm{TiO}}_{2}\right)}_{n}$ (with n = 1–10) are promising photocatalysts for ${\mathrm{CO}}_{2}$ conversion; however, their size‐dependent stability and reactivity are not fully characterized. This study uses density functional theory (M06/def2‐TZVP) and global and local reactivity descriptors to identify “magic number” clusters that exhibit high stability. The stability function ($\varepsilon^{3}$), reveals n = 2, 4, and 8 as magic numbers. Electrophilicity analysis (Δω) shows moderate electrophilicity for n = 1–5 and strong electrophilicity for n = 7–10, while the magic numbers display reduced reactivity. Fukui functions and fractional occupation number‐weighted density ($N_{\mathrm{FOD}}$) highlight localized reactivity. Notably, they reveal n = 6 to be highly electrophilic, with distinct “hot” electron sites. ${\mathrm{CO}}_{2}$ interaction energies inversely correlate with cluster stability: unstable clusters (n = 3, 5, and 9) strongly bind ${\mathrm{CO}}_{2}$ (up to 0.72 eV), while magic numbers weakly physisorb it (e.g., 0.45 eV for n = 8). Non‐covalent interaction (NCI) analysis confirms Ti–OCO attraction and C‐repulsive sites. Together, these results establish design principles for ${\mathrm{TiO}}_{2}$ cluster catalysts that balance stability with tailored reactivity for ${\mathrm{CO}}_{2}$ activation.

## Graphical abstract

The analysis of the global and local stability and global and local reactivity of (TiO_2_)_*n*_, (*n* = 1–10) clusters and the study of their magic numbers in relation to CO_2_ are examined. Additionally, we characterize this interaction.

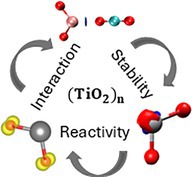

## 1 Introduction

In industrial and laboratory settings, clusters serve as versatile scaffolds for the synthesis of nanomaterials composed of a small number of atoms with well‐defined positions [1, 2]. Thus, research in theoretical and experimental fields, in this sense, transition metal oxide clusters are particularly important due to their applicability in several technological and scientific domains, ranging from energy conversion and storage to environmental remediation, sensors and optoelectronic devices [3]. As an example, extensive studies have been conducted both theoretically [4, 5] and experimentally [6, 7] towards ${\left({\mathrm{TiO}}_{2}\right)}_{n}$ clusters.

The structures of ${\left({\mathrm{TiO}}_{2}\right)}_{n}$ clusters are well characterized, with most of these structures exhibiting high stability. Owing to this stability property, they are extensively employed as photocatalysts [8, 9, 10, 11], particularly in the photoreduction of ${\mathrm{CO}}_{2}$ [12, 13, 14]. This makes them a promising candidate for the production of renewable energy sources and the mitigation of global warming [12, 15, 16, 17]. However, the stability of ${\left({\mathrm{TiO}}_{2}\right)}_{n}$ clusters can vary significantly depending on the number of atoms [18].

Titanium dioxide (${\mathrm{TiO}}_{2}$) occurs naturally in three major crystallographic phases: rutile, anatase and brookite, being rutile the most thermodynamically stable phase at 0 K and zero pressure [19, 20, 21]. However, for structures smaller than 14 nm, anatase exhibits greater stability than rutile [4, 22]. As these size scales continue to decrease, it is not sufficient to assume that the electronic structure properties remain unchanged [23], as they vary consistently with volume. In this context, understanding their physicochemical properties, the electronic structure of ${\left({\mathrm{TiO}}_{2}\right)}_{n}$ clusters and how it changes over the variation of *n*, is crucial for the potential conversion of ${\mathrm{CO}}_{2}$ into more valuable chemicals, such as HCOOH and ${\mathrm{CH}}_{4}$ [24, 25, 26, 27].

Thermodynamic and kinetic stability are size‐dependent properties; specific cluster structures are expected to be favored, known as “magic numbers.” Previous studies have characterized ${\mathrm{TiO}}_{2}$ clusters based on their sizes, identifying global minima and evaluating basic stability properties of these clusters [2, 28]. However, there remains a substantial gap in the understanding of the electronic structure and the characterization of the “magic numbers” of ${\left({\mathrm{TiO}}_{2}\right)}_{n}$ clusters, as well as how these clusters interact with ${\mathrm{CO}}_{2}$. Thus, this study investigates the properties of clusters with sizes varying from n=1 to 10. The study analyzed stability parameters, including the global stability ranking function ($\varepsilon^{3}$) [29], which incorporates quantities derived from electronic structure calculations (e.g., ionization potential and reaction spontaneity via Gibbs energy) the global reactivity parameter (Δω) [30], which determines the nucleophilic or electrophilic character of each cluster; Fukui function analyses ($f^{-}\left(r\right)$ and $f^{+}\left(r\right)$) [31], which reveal the inherent tendency to donate or accept electrons for each cluster; and Fractional Occupation Number Weighted Density (FOD) analyses, which locate the “hot” electrons within the structures. After performing these cluster analyses, we calculated the interactions between each cluster and ${\mathrm{CO}}_{2}$, identifying regions of maximum and minimum interaction. We characterized the primary adsorption sites and evaluated the nature of this interaction using indices that identify and quantify weak, non‐covalent interactions.

## 2 Computational Details

The cluster structures studied here were based on the work of Neogi and Chaudhury [2], who explored different local minima associated with ${\mathrm{TiO}}_{2}$ conformers. In the present work, we focus exclusively on the global minimum structure for each ${\left({\mathrm{TiO}}_{2}\right)}_{n}$ cluster, with *n* ranging from 1 to 10. To refine these geometries and calculations of vibrational frequencies, in this work Density Functional Theory (DFT) [32, 33, 34] is employed within a hybrid meta‐GGA approximation, M06 functional [35], with def2‐TZVP basis sets [36], considering both singlet and triplet spin multiplicities for each structure, in the Gaussian 09 software [37]. All analyses in which the methodology was modified are reported in their respective sections.

In this study, CASPT2 calculations were considered to evaluate the energies of the clusters. For the smaller clusters, we found that the singlet‐triplet separation and ionization energies obtained with CASPT2 and the methodology employed here differ by approximately 10%. Due to the high computational cost associated with treating larger clusters, we opted to use the M06 functional combined with a triple‐zeta basis set (def2‐TZVP).

The following subsections summarize the parameters employed to evaluate the structural and electronic properties of ${\left({\mathrm{TiO}}_{2}\right)}_{n}$ (n=1−10) clusters.

### 2.1 Stability and Reactivity Functions

The stability ranking function ($\varepsilon^{3}$) (Equation 2) is a classification metric for stability that can distinguish magic numbers according to experimental results and has already been shown to be effective in identifying magic numbers across different groups of the periodic table, including clusters of ${\mathrm{Si}}_{n}$ [38, 39, 40, 41], clusters of ${\mathrm{Mg}}_{n}$ [29, 42, 43], clusters of ${\mathrm{Na}}_{n}$ [44], among others.

(1) $$\varepsilon^{3}=\left|\mathrm{IP}\right|\times \left|E_{S-T}\right|\times \left|\Delta G_{\text{atom},298K}^{\circ }\right|$$

All three terms in Equation (2) are determined directly from the wavefunction and the electronic density. The first term represents the ionization potential (IP), analogous to the HOMO energy within the Koopmans' approximation, obtained from the energy difference between the neutral cluster and its corresponding cation. The second term corresponds to the energy associated with the singlet–triplet excitation gap ($E_{S-T}$). Finally, the third term refers to the Gibbs free energy of atomization ($\Delta G_{\text{atom},298K}^{\circ }$), which expresses the spontaneity of cluster formation (Equation 2). Low values of $\varepsilon^{3}$ indicate low cluster stability, whereas high values denote high stability, defining the so‐called magic numbers [29, 38].

(2) $$\Delta G_{\text{atom},298K}^{\circ }=-\left(G_{\text{cluster},298K}^{\circ }-\left({N_{\text{atoms}}}^{*}G_{\text{atom},298K}^{\circ }\right)\right)$$

The reactivity of a molecule can be well defined using qualitative and quantitative parameters that help to predict the behavior of a given chemical reaction [30, 45, 46]. The electrophilicity index ω evaluates the electrophilic power of a species and has been successfully used to rationalize chemical reactions [47], determine aromatic bonding [48], define reactions in multielectronic species [49], among other applications [50]. It is defined as:

(3) $$\omega =\frac{\mu^{2}}{2\eta }$$

where the term μ is the electronic chemical potential and η is the total hardness of the system [51]. From Equation (3), were derived two new index: the electro‐donating power ($\omega^{-}$), and the electro‐accepting power ($\omega^{+}$), given by Equations (4) and (5), respectively.

(4) $$\omega^{-}=\frac{{\left(\mu^{-}\right)}^{2}}{2\eta }\equiv \frac{{\left(3\mathrm{IP}+\mathrm{EA}\right)}^{2}}{16\left(\mathrm{IP}-\mathrm{EA}\right)}$$

(5) $$\omega^{+}=\frac{{\left(\mu^{+}\right)}^{2}}{2\eta }\equiv \frac{{\left(\mathrm{IP}+3\mathrm{EA}\right)}^{2}}{16\left(\mathrm{IP}-\mathrm{EA}\right)}$$

The molecule can present both behaviors, depending on the species with which it is interacting, both index were combined to evaluate the electrophilicity of the system, by the net electrophilicity ($\Delta \omega^{\pm }$) [47, 50, 52, 53] represented by Equation (6).

(6) $$\Delta \omega^{\pm }=\omega^{+}-\frac{1}{\omega^{-}}$$

where $\Delta \omega^{\pm }$ below 0.59 indicate nucleophilic structures, values between 0.59 and 1.29eV indicate moderate electrophile and values, greater than 1.29eV a strong electrophile.

Besides analyzing the stability and reactivity of the clusters, we have also evaluated their interaction with ${\mathrm{CO}}_{2}$. To evaluate the interaction between ${\left({\mathrm{TiO}}_{2}\right)}_{n}$ clusters and the ${\mathrm{CO}}_{2}$ molecule, the net reactivity index ($\Delta \omega_{R}^{\pm }$) [50], defined in Equation (7) can also be used.

(7) $$\Delta \omega_{R}^{\pm }=\omega^{+}\left(\text{most electrophile}\right)-\frac{1}{\omega^{-}\left(\text{less electrophile}\right)}$$

The parameters necessary for evaluating stability and reactivity were obtained by calculating the neutral singlet and triplet states, as well as the cation and anion using the M06 functional and the def2‐TZVP basis set.

### 2.2 Local Extensions and Site Selectivity—Fukui

To analyze the kinetic stability of the molecular systems and describe the site selectivity in a molecule we have used a local reactivity descriptor based on the Fukui ($f^{+}\left(r\right)$ and $f^{-}\left(r\right)$) [31, 54] indices. The local density functional quantity is the Fukui function.

(8) $$f\left(r\right)={\left(\frac{\partial \rho \left(r\right)}{\partial N}\right)}_{v\left(r\right)}$$

The Fukui function shows how the number of incoming or outgoing electrons is spatially redistributed in a molecule. Hence, it is of primary importance to chemistry. For a finite system, the derivative in 8 is discontinuous and all derivatives with respect to the number of electrons are discontinuous. Parr and Yang proposed associating $f\left(r\right)$ with reactivity indices. In nucleophilic attack (the increasing number of electrons) [55]:

(9) $$f^{+}\left(r\right)={\left(\frac{\partial \rho \left(r\right)}{\partial N}\right)}_{v\left(r\right)}^{+}$$

and in an electrophilic attack (decreasing number of electrons),

(10) $$f^{-}\left(r\right)={\left(\frac{\partial \rho \left(r\right)}{\partial N}\right)}_{v\left(r\right)}^{-}$$

In the finite difference approach $f^{-}\left(r\right)$ and $f^{+}\left(r\right)$ reduce to the original Fukui indices [31, 47, 55, 56, 57].

(11) $$f^{+}\left(r\right)\approx \rho_{N+1}\left(r\right)-\rho_{N}\left(r\right)\;\text{for nucleophilic attack}$$

(12) $$f^{-}\left(r\right)\approx \rho_{N}\left(r\right)-\rho_{N-1}\left(r\right)\;\text{for electrophilic attack}$$

where $\rho_{N}$, $\rho_{N+1}$, and $\rho_{N-1}$ are electron density functions of N, N+1, and N−1 electron systems, respectively, at the same geometry.

Integrating the information contained in 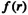 around a given atom A can be approximated using the gross atomic charge $q_{k}$. from a Mulliken population analysis. The condensed Fukui function index on atom *k* is:

(13) $$f^{+}\left(r\right)=\left[q_{k}\left(N+1\right)-q_{k}\left(N\right)\right]\;\text{for nucleophilic attack}$$

(14) $$f^{-}\left(r\right)=\left[q_{k}\left(N\right)-q_{k}\left(N-1\right)\right]\;\text{for electrophilic attack}$$

For the $f^{-}$ cationic and $f^{+}$ anionic, the SCh‐Kohn‐Sham (DFJ‐RKS) method [58] was employed in combination with the MN15‐L functional [59] and the def2‐TZVP basis set. For the electron density difference‐based $f^{-}\left(r\right)$ and $f^{+}\left(r\right)$, the SCh‐Kohn‐Sham (DFJ‐RKS) method was employed in combination with the M06 functional, and the def2‐SVP basis set, using the MOLPRO program [60, 61].

### 2.3 Fractional Occupation Number Weighted Density—$N_{\mathrm{FOD}}$

The Fractional Occupation Number Weighted Density ($N_{\mathrm{FOD}}$) is an extensive property that can be used to analyze the location of “hot” electrons, those chemically active and strongly correlated within the structure. $N_{\mathrm{FOD}}$ was carried out as a general theoretical diagnostic for the electronic structure of the systems [62, 63]. To obtain the $N_{\mathrm{FOD}}$ values, it is first necessary to perform an electronic density functional calculation using the optimized geometry, followed by the construction of a fractional density that accounts only for the contribution of strongly correlated electrons. The correlation between $N_{\mathrm{FOD}}$ values and experimental reactivity data reveals a stable system for values below 1. In the present study, this calculation was performed using the M06 functional with the def2‐TZVP basis set. To convert this property into an intensive analysis, the total $N_{\mathrm{FOD}}$ value for each cluster was normalized by the number of atoms. The $N_{\mathrm{FOD}}$ calculations were performed using the ORCA 6.0 [64] software, following the temperature‐related conventions. The M06 functional, which includes 27% Hartree–Fock exchange, was employed in accordance with standard protocols [65, 66].

### 2.4 Non‐Covalent Interactions Index

The non‐covalent interaction (NCI) [67, 68], a visualization index based on electron density (ρ) and the reduced density gradient ($s\left(r\right)$), is a topological tool that reveals and qualitatively characterizes the nature of molecular interactions as attractive or repulsive through isosurfaces with a color code indicating the interaction type [69, 70, 71]. This method combines the RDG with the NCI index to visualize and characterize non‐covalent interactions in three‐dimensional space. Given by:

(15) $$s\left(r\right)=\frac{\left|\nabla \rho \left(r\right)\right|}{C_{F}\rho {\left(r\right)}^{4/3}}$$

where $C_{F}=2{\left(3\pi^{2}\right)}^{1/3}$, $\rho \left(r\right)$ is the electron density and $\nabla \rho \left(r\right)$ is its first derivative. The scatterplot of sign ($\lambda_{2}$) ρ (a.u.) (the signed electron density multiplied by the second eigenvalue of the electron density Hessian) versus the reduced density gradient (RDG) illustrates non‐covalent interactions (NCIs) within the examined system. The RDG on the *y*‐axis identifies regions with a low electron density gradient, and the *x*‐axis, sign ($\lambda_{2}$) ρ, distinguishes the interaction types. In this work, the NCI calculations were performed using the geometry optimized at the M06/def2‐TZVP level.

### 2.5 Quantum Theory of Atoms in Molecules (QTAIM)

Topological data of clusters ${\left({\mathrm{TiO}}_{2}\right)}_{n}$ with *n* = 1–10 interacting with ${\mathrm{CO}}_{2}$, were described by the Quantum Theory of Atoms in Molecules (QTAIM). Electron density ($\rho \left(r\right)$), Laplacian electron density ($\nabla^{2}\rho \left(r\right)$), kinetic energy density (*G*(*r*)), potential energy density (*V*(*r*)), and total electron density (*H*(*r*) = *G*(*r*) + *V*(*r*)) [72, 73, 74, 75] were used to evaluate molecular interactions at the Binding Critical Point (BCP). The QTAIM study was performed using AIMALL software [76].

## 3 Results and Discussion

### 3.1 Stability Ranking Function ($\varepsilon^{3}$) and Electrophilicity Indices (Δω)

**FIGURE 1:**
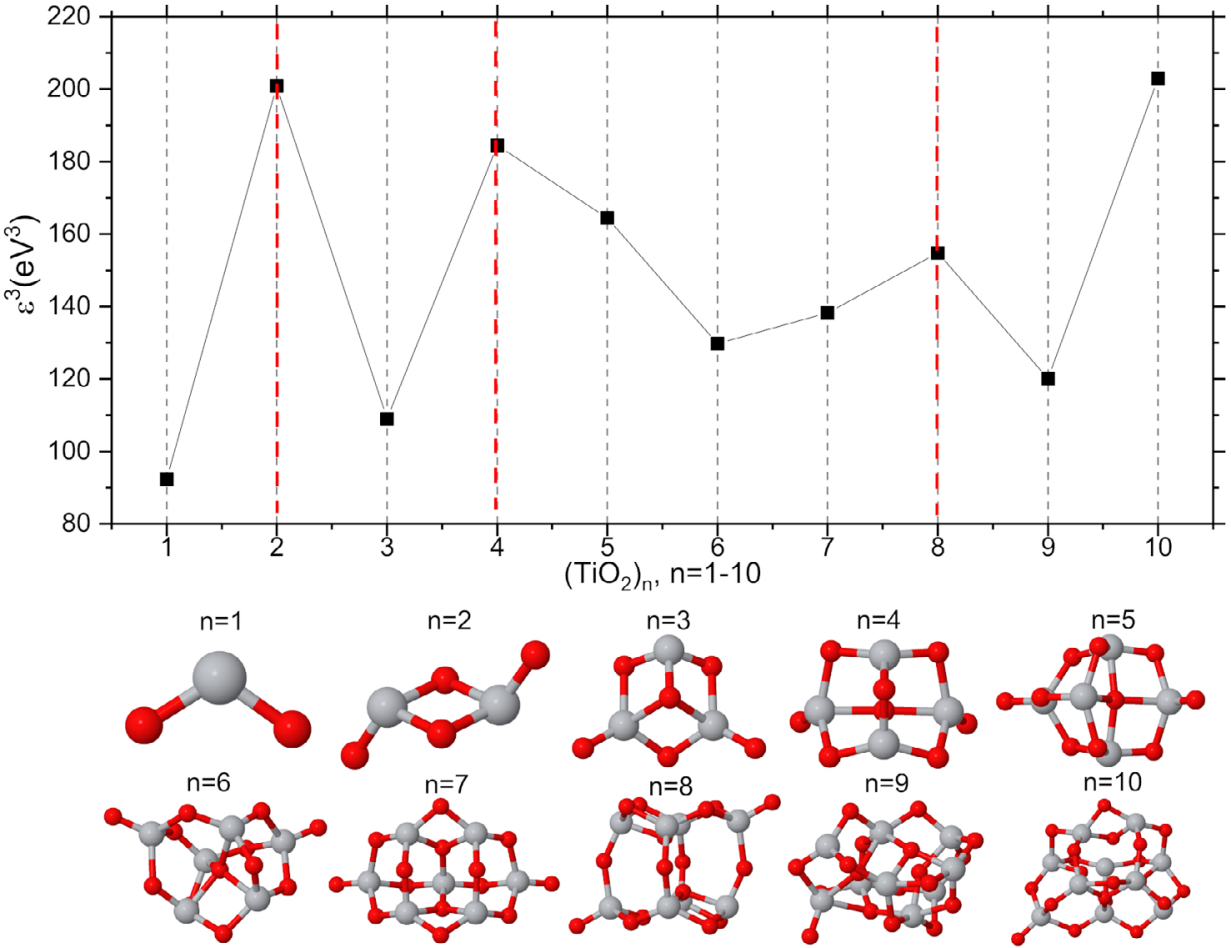
Stability ranking function ($\varepsilon^{3}$ in ${\mathrm{eV}}^{3}$) for ${\left({\mathrm{TiO}}_{2}\right)}_{n}$ clusters, with *n* ranging from 1 to 10. The optimized geometries (M06/def2‐TZVP) of the clusters are also shown in the graph.

Figure 1 shows the Stability Ranking Function ($\varepsilon^{3}$) values correlated with ${\left({\mathrm{TiO}}_{2}\right)}_{n}$ clusters as a function of their size. The structural representation of each cluster is also shown.

Clusters with n = 2, 4, and 8 are remarkably stable compared to neighboring clusters. The n = 10 system also displays high stability relative to the n = 9 cluster. However, due to the absence of data for the n = 11 cluster, its relative stability among adjacent clusters cannot be conclusively determined. According to Syzgantseva et al. [28], ${\left({\mathrm{TiO}}_{2}\right)}_{n}$ clusters with n = 5, 7, and 9 tend to be less stable. This trend aligns with $\varepsilon^{3}$, indicating that odd n (1, 3, 5, 7, 9) are energetically less favorable. While no universal rule explains cluster stability, the presented analyses suggest the clusters with even n = 2, 4, and 8 potential magic numbers. The n = 6 cluster deviates from this pattern. Despite being an even number, it exhibits reduced stability compared to adjacent clusters and emerges as a potentially reactive species. To analyze the $\varepsilon^{3}$ values, as well as each of its component parameters, refer to Table S1 in the Supporting Information (SI).

**FIGURE 2:**
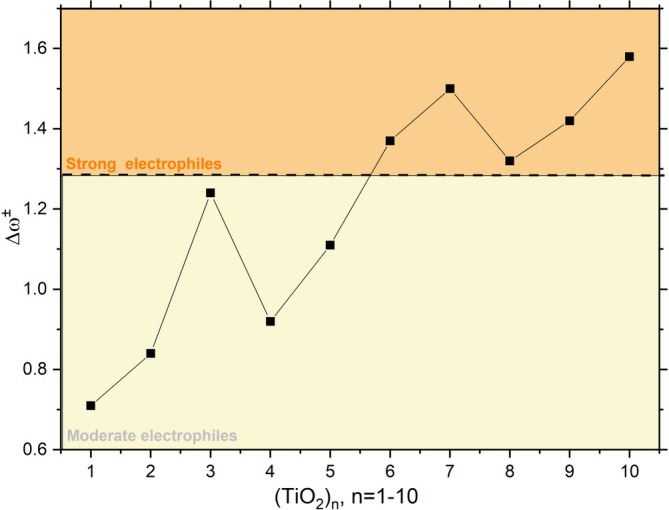
Electrophilicity indices ($\Delta \omega^{\pm }$) applied to ${\left({\mathrm{TiO}}_{2}\right)}_{n}$ clusters, with n varying from 1 to 10. The graph is divided into two sections: moderate electrophiles ($0.59<\Delta \omega^{\pm }<1.29$—highlighted in yellow), and strong electrophiles ($\Delta \omega^{\pm }>1.29$—highlighted in orange).

A stable structure should exhibit low reactivity, indicating resistance to bond formation. Figure 2 presents the electrophilicity indices $\Delta \omega^{\pm }$ for the ${\left({\mathrm{TiO}}_{2}\right)}_{n}$ clusters. Structures with values below $\Delta \omega^{\pm }<0.59\;\mathrm{eV}$ are characterized as nucleophilic.

Based on $\Delta \omega^{\pm }$, the clusters with n = 1, 2, 3, 4, and 5 can be classified as moderate electrophiles, while clusters with n = 7, 8, 9, and 10 are classified as strong electrophiles. Since none of the structures exhibited values lower than 0.59, none of them can be characterized as nucleophilic. Interestingly, although n = 8 is classified as an electrophile, its lower $\Delta \omega^{\pm }$ value compared to the other values in this group indicates reduced in its attraction for electrons, therefore a lower capacity to form certain chemical bonds. A similar pattern is evident for n = 2 and 4, which are identified as potential magic numbers in agreement with the $\varepsilon^{3}$ values, exhibiting notable stability and reduced reactivity compared to their neighbors. The complete list of the parameters used for $\Delta \omega^{\pm }$ values are given in Table S2.

In contrast, the n = 3 cluster, which is within the moderate electrophile group, displays higher reactivity than adjacent clusters, which aligns with its stability parameter. The n = 10 cluster, which is both stable and a strong electrophile, exhibits the highest reactivity value. This dual property makes it particularly interesting for ${\mathrm{CO}}_{2}$ catalysis and photocatalysis because an effective catalyst must have sufficient reactivity to interact with target substrates and structural stability to prevent disintegration during reactions, see Table S2 in SI. The global stability ($\varepsilon^{3}$) and global reactivity (Δω) parameters provide an overall analysis of the cluster.

### 3.2 Fukui Function $f^{-}\left(r\right)$ and $f^{+}\left(r\right)$

**FIGURE 3:**
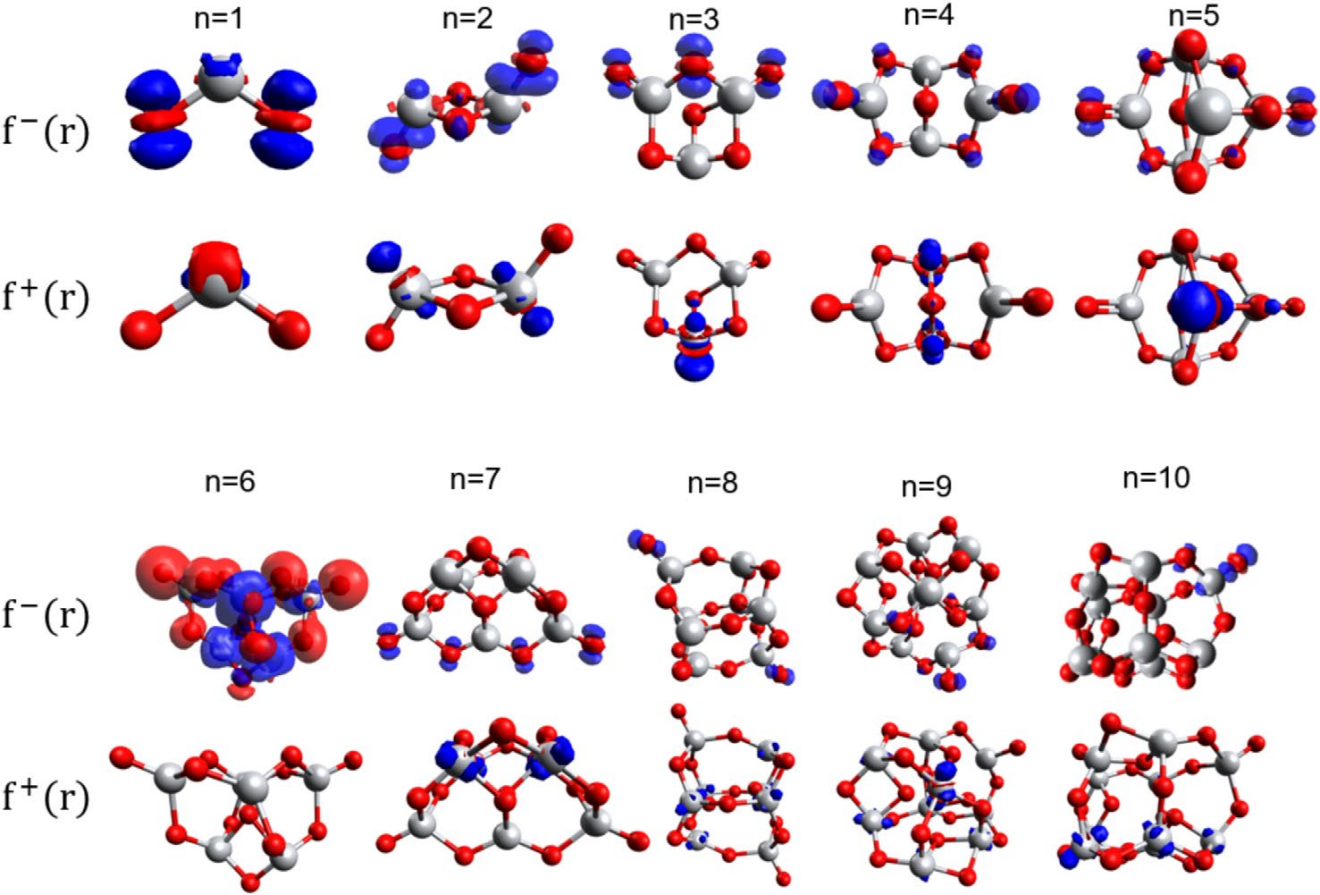
Fukui functions $f^{-}\left(r\right)$ and $f^{+}\left(r\right)$ for the cluster ${\left({\mathrm{TiO}}_{2}\right)}_{n}$, where red indicates regions of lower electron density, while blue represents areas of electronic redistribution within the molecule.

To study the chemical reactivity of compounds and the selectivity at a given site, local descriptors can be used, directing applications, thus the Fukui function $f^{-}\left(r\right)$ and $f^{+}\left(r\right)$ are represented in Figure 3 which highlights the electronic distribution within the molecule.

Analysis of the $f^{-}\left(r\right)$ values reveals that clusters with n = 1, 2, and 6 have the highest electron density, while clusters with n = 4, 5, 7, 8, 9, and 10 have lower $f^{-}\left(r\right)$ densities. Regarding $f^{+}\left(r\right)$, only the clusters with n = 1 and 3 display a small density localized on their Ti atoms, while the others exhibit negligible values. Notably, the cluster with n = 6 stands out again, as it presents low kinetic stability, behaves as an electrophile, and shows the highest $f^{-}\left(r\right)$ density of all the analyzed clusters. The Fukui values for each cluster atom are shown in Tables S3–S12 in the SI.

These results suggest that the oxygen in the clusters can react with other molecules through electrophilic attacks. Conversely, the sites that undergo nucleophilic attacks in the clusters are Ti.

### 3.3 $N_{\mathrm{FOD}}$ Parameter

**FIGURE 4:**
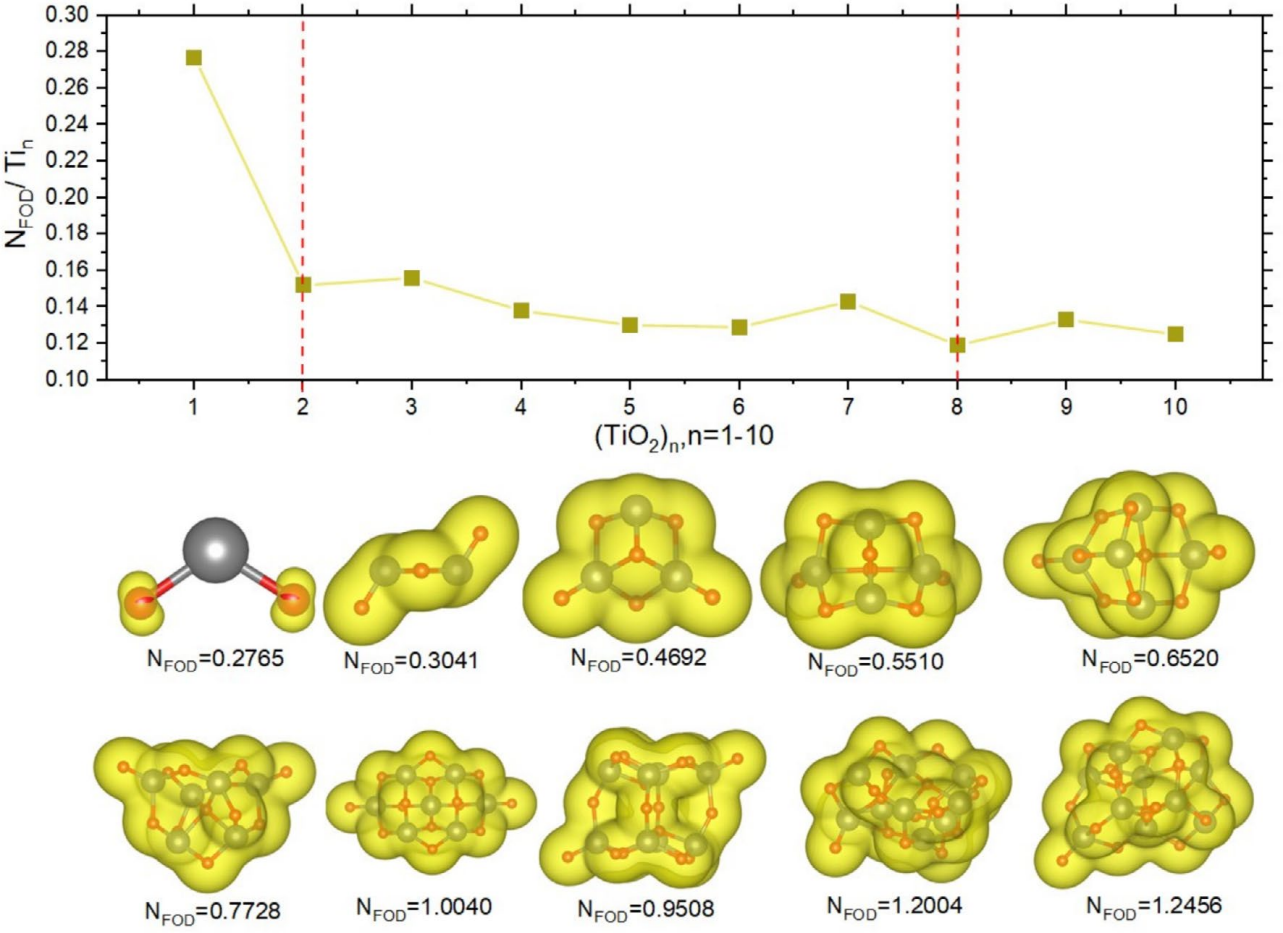
$N_{\mathrm{FOD}}$ values are shown below each cluster as an extensive property, while the graph displays the corresponding atomic intensive property.

Figure 4 shows the total $N_{\mathrm{FOD}}$ values (representing the entire cluster as an extensive property), while the accompanying graph shows the normalized values per atom (intensive property). In addition, the electronic density is visually represented in yellow, with the isosurface set consistently at 0.005 across all analyses.

Analyzing the electronic density values, one can observe that, for n = 1, the hot electrons are localized on the oxygen atoms. In larger clusters, this density becomes more dispersed due to structural rearrangements, which place the oxygen atoms in different positions. The extensive $N_{\mathrm{FOD}}$ values, represent the total number of hot electrons for each cluster. This value tends to increase with cluster size, with the highest value observed for n = 10 and the lowest for n = 1. From n = 7 to 10, the $N_{\mathrm{FOD}}$ values exceed 1, except for n = 8, which is the only cluster among n = 7, 8, 9, and 10 that does not surpass this threshold.

Examining the intensive $N_{\mathrm{FOD}}$, obtained by dividing the total $N_{\mathrm{FOD}}$ by the number of Ti, it was noted that n = 2 and n = 8 exhibit a decrease in their values compared to neighboring clusters, indicating lower hot electron densities. This result is consistent with other analyses presented in this study, which identify n = 2 and n = 8 as the most stable clusters and, consequently, those with the fewest hot electrons. Clusters n = 3, 7, and 9 show higher intensive behavior compared to their neighbors, indicating greater hot electron density. In contrast, n = 6 maintains a lower value relative to its adjacent clusters.

Considering the analyses of $N_{\mathrm{FOD}}$ and the Fukui densities presented in Section 3.2, the cluster with n = 6 exhibits a high $f^{-}\left(r\right)$ density along with a low $N_{\mathrm{FOD}}$ value. This suggests the presence of a nucleophilic site within an otherwise electronically stable system. Such behavior indicates localized reactivity without significant delocalization of hot electrons across the entire cluster. In contrast, the n = 7 cluster displays the opposite trend: a low $f^{-}\left(r\right)$ density combined with a high $N_{\mathrm{FOD}}$ value, which may reflect a globally unstable electronic configuration or a tendency toward excitation. However, the absence of a clearly defined nucleophilic site, due to the spread and low intensity of the $f^{-}\left(r\right)$ density across multiple atoms, implies a more diffuse distribution of reactivity.

### 3.4 Interactions Between ${\left({\mathrm{TiO}}_{2}\right)}_{n}$ Clusters and ${\mathrm{CO}}_{2}$

Considering all the analyses of stability and reactivity of the clusters, their interaction with a catalytically relevant molecule was evaluated in order to identify the sites of maximum interaction and verify whether the clusters maintained their stability and reactivity trends in the presence of another molecule. The structure analyzed was ${\mathrm{CO}}_{2}$, due to its extensive interest in reduction processes in recent years. The geometries associated with the sites of maximum and minimum interaction can be found in the SI. The interaction energy calculations between the cluster and ${\mathrm{CO}}_{2}$ were performed at the M06/def2‐TZVP level, except for n = 10, for which the geometry optimization was carried out using the M06/def2‐SV(P) level of theory. This choice was made due to the high computational cost of employing a triple‐zeta basis set for a cluster with a larger number of atoms. However, once the optimized geometries were obtained, single‐point energy calculations were performed using the def2‐TZVP basis set to ensure consistency in the interaction energy analysis.

**FIGURE 5:**
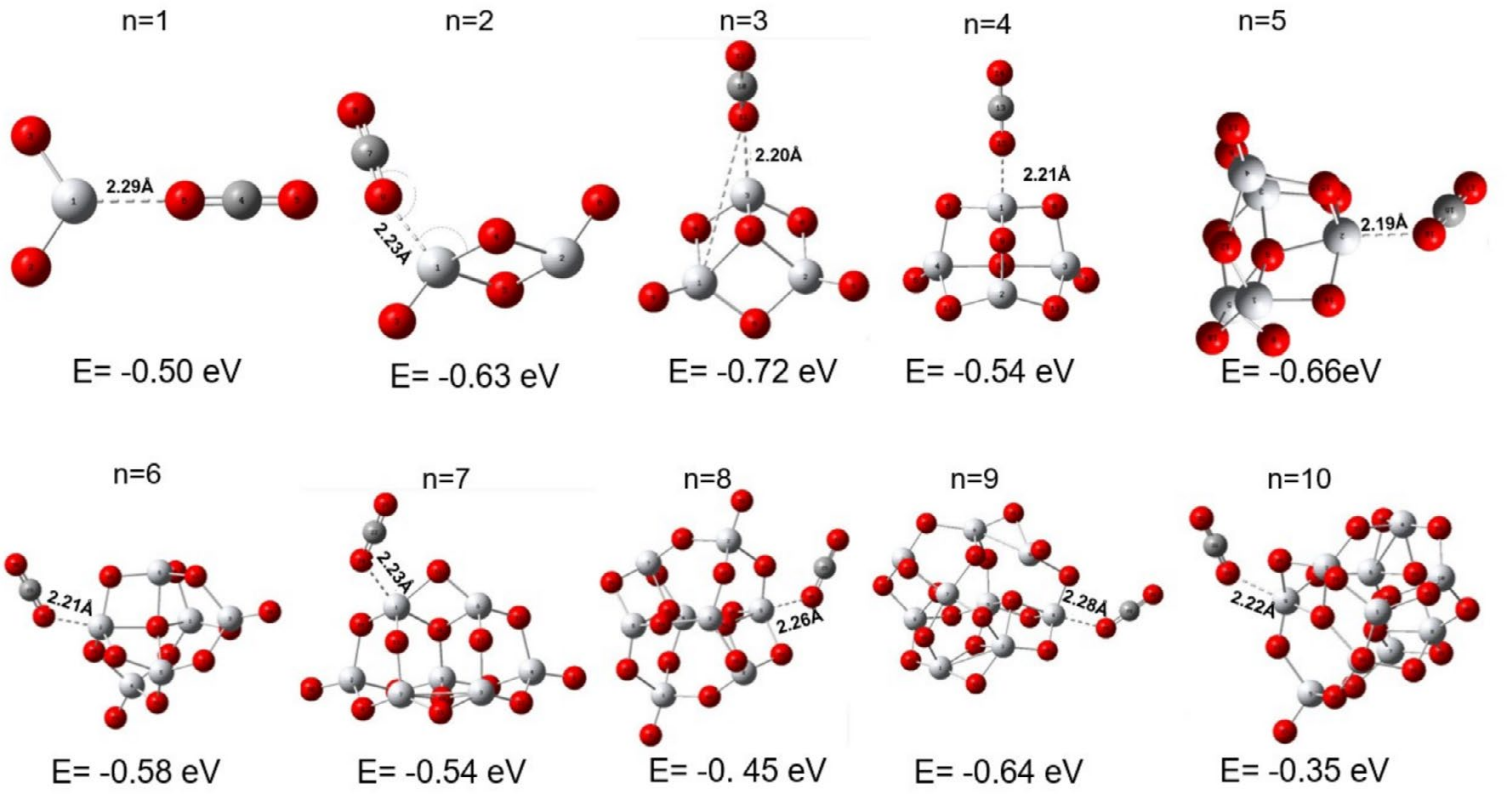
Values assigned to the interaction energy (eV) between the ${\left({\mathrm{TiO}}_{2}\right)}_{n}$ clusters and ${\mathrm{CO}}_{2}$, considering the site of maximum interaction.

Different interaction sites between ${\mathrm{CO}}_{2}$ and each ${\left({\mathrm{TiO}}_{2}\right)}_{n}$ clusters were identified. It is expected that stable clusters exhibit weak interactions, while reactive clusters form stronger interactions with the gas. The interaction of ${\mathrm{CO}}_{2}$ with each of the titanium atoms exposed on the cluster surface was tested as well as with the oxygen atoms. When positioned near the oxygens, a repulsive trend was observed due to the oxygen in ${\mathrm{CO}}_{2}$, but when ${\mathrm{CO}}_{2}$ was approached to the titanium atoms in the clusters, a minimum was found, and the interaction energy varied depending on the specific Ti site in the cluster. The interaction energies and the adsorption sites are presented in Figure 5.

**FIGURE 6:**
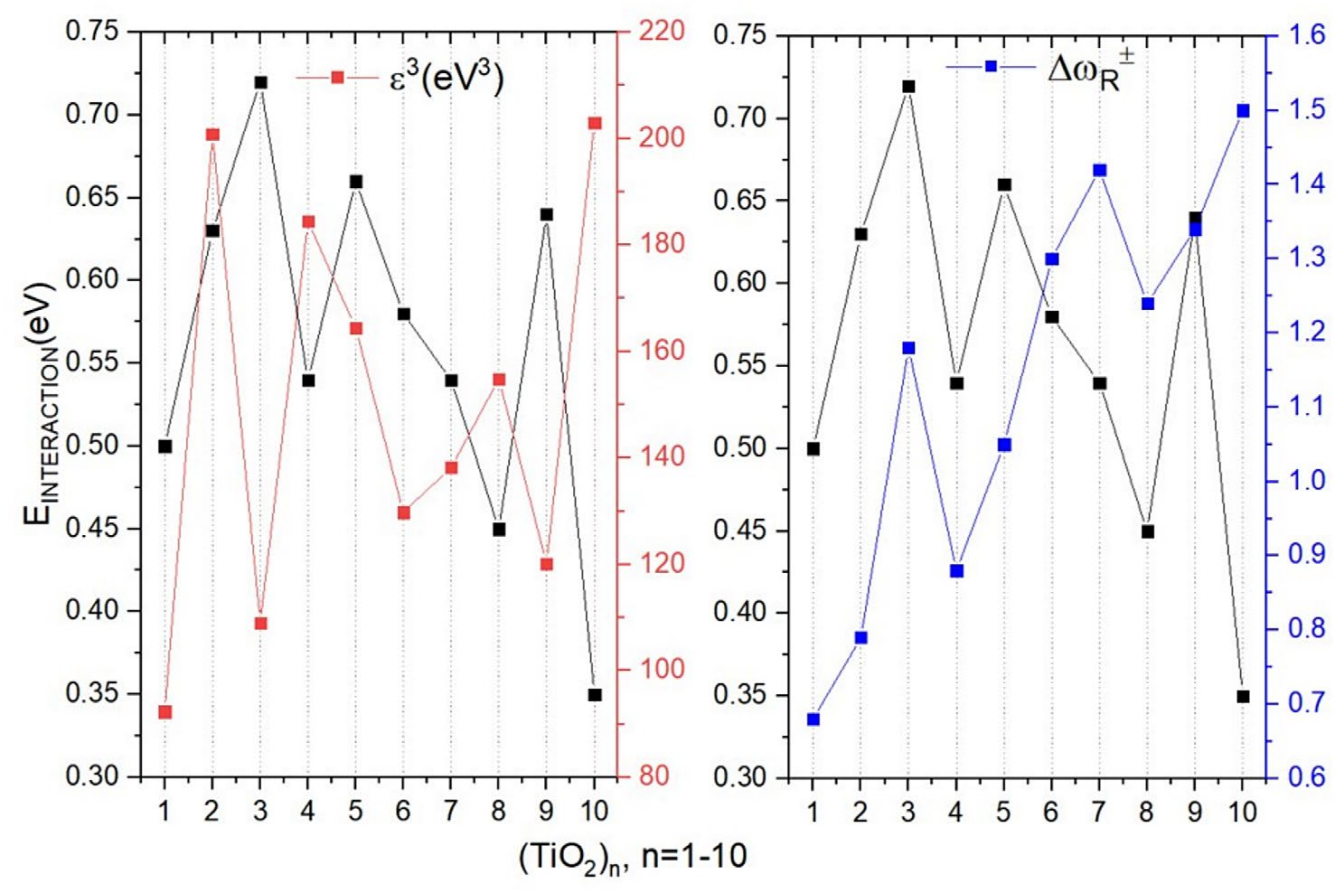
(a) Values of the stability ranking function ($\varepsilon^{3}$) (red *y*‐axis) with the interaction energy of the cluster of ${\left({\mathrm{TiO}}_{2}\right)}_{n}$ with ${\mathrm{CO}}_{2}$ (black *y*‐axis); (b) values of the global relative electrophilicity index $\Delta \omega_{R}^{\pm }$ (blue *y*‐axis) with the interaction energy of the cluster of ${\left({\mathrm{TiO}}_{2}\right)}_{n}$ with ${\mathrm{CO}}_{2}$ (black *y*‐axis).

Based on the maximum interaction sites between ${\mathrm{TiO}}_{2}$ clusters and ${\mathrm{CO}}_{2}$, we can assess the stability and reactivity of these clusters. The lowest interaction energies are observed for the clusters n = 4 (−0.54 eV), n = 8 (0.45 eV), and n = 10 (−0.35 eV), compared to their neighboring clusters. In contrast, the highest interaction sites are found for n = 3 (−0.72 eV), n = 5 (−0.66 eV), and n = 9 (−0.64 eV). Interestingly, clusters with lower interaction energies, such as n = 4 and n = 8, are identified in this work as magic numbers. The cluster with n = 10 could not be classified as such due to the absence of neighboring references. To analyze the descriptors of the individual clusters and their relationship with the cluster–${\mathrm{CO}}_{2}$ system, we compiled in Figure 6 the stability ranking function $\varepsilon^{3}$ values (red *y*‐axis) and the $\Delta \omega^{\pm }$ values (blue *y*‐axis) with the interaction energies of the ${\mathrm{TiO}}_{2}$ cluster with ${\mathrm{CO}}_{2}$ (always at the site of maximum interaction) (black *y*‐axis). The $\Delta \omega^{\pm }$ values indicate the relative reactivity of each cluster in relation to ${\mathrm{CO}}_{2}$.

The ${\mathrm{CO}}_{2}$ exhibited the lowest value of $\omega^{-}$ among all species analyzed, it was considered the reference electrophile in the calculation of the global relative electrophilicity index. Thus, the relative index was defined as:

(16) $$\Delta \omega_{R}^{\pm }=\omega^{+}\left({\left({\mathrm{TiO}}_{2}\right)}_{n}\right)-\frac{1}{\omega^{-}\left({\mathrm{CO}}_{2}\right)}$$

This equation provides a quantitative measure of the cluster's electrophilic behavior relative to ${\mathrm{CO}}_{2}$, where higher values of $\Delta \omega_{R}^{\pm }$ indicate a greater tendency for the cluster to act as an electrophile in the presence of ${\mathrm{CO}}_{2}$.

A correlation between the global stability parameter and the interaction energy of the cluster with ${\mathrm{CO}}_{2}$ is observed. Clusters exhibiting higher stability compared to their neighbors, such as n = 4 and n = 8, display lower interaction energy values when compared to adjacent clusters. This occurs because these properties are inversely proportional, more stable clusters tend to resist interactions with other structures.

Conversely, clusters with higher interaction energies, such as n = 3, n = 5, and n = 9, also exhibit significantly higher reactivity, indicating a greater tendency to form bonds. In contrast, clusters characterized by low interaction energies, like n = 2, n = 4, n = 8, and n = 10, show low reactivity, suggesting resistance to interactions with other structures.

A NCI analysis was performed to qualitatively characterize the interactions between the cluster and ${\mathrm{CO}}_{2}$ after considering the interaction energies and geometry obtained at the highest global interaction energy. This analysis is based on the empirical observation that non‐covalent interactions are associated with regions of low electron density and small reduced density gradient values.

**FIGURE 7:**
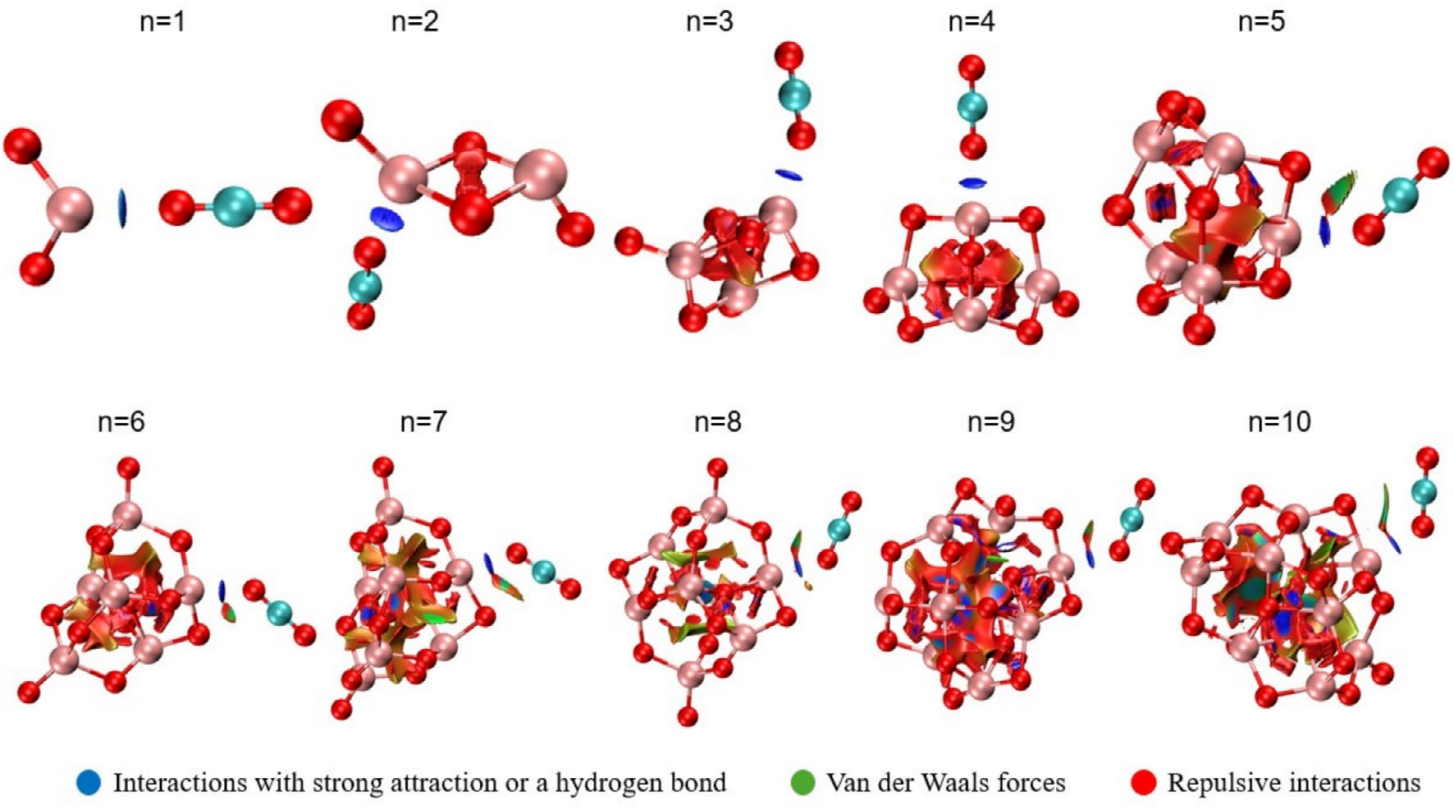
NCI representation in 3D for the ${\left({\mathrm{TiO}}_{2}\right)}_{n}$ clusters interacting with ${\mathrm{CO}}_{2}$ at the site of maximum interaction. Calculated with M06/def2‐TZVP.

**FIGURE 8:**
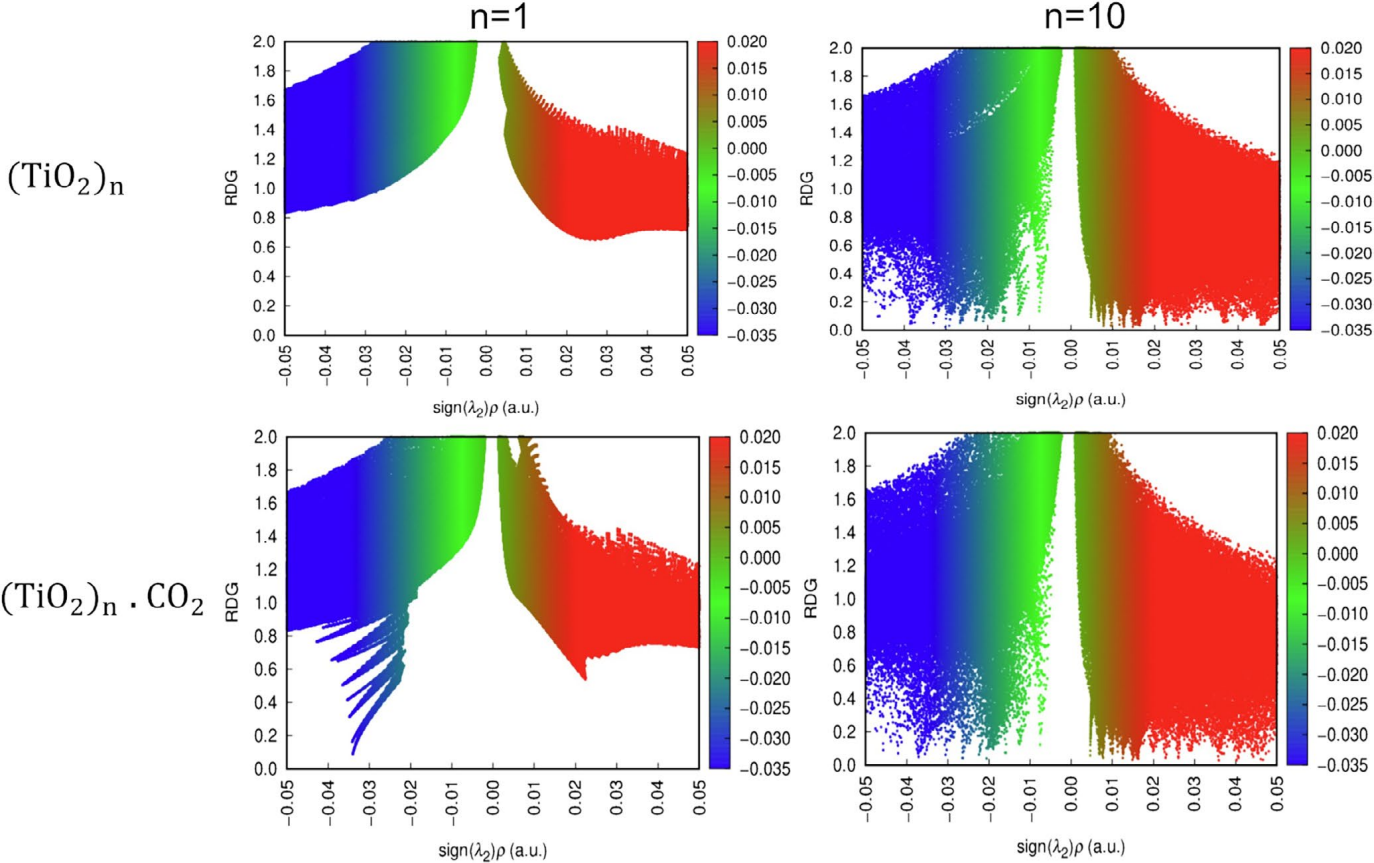
2D visualization of non‐covalent interactions (NCI) between ${\mathrm{CO}}_{2}$ and ${\left({\mathrm{TiO}}_{2}\right)}_{n}$ clusters at optimal adsorption sites and only ${\left({\mathrm{TiO}}_{2}\right)}_{n}$. Isosurfaces are colored according to s sign($\lambda_{2}$) ρ (a.u.) values: blue indicates strong attractive interactions, green denotes weak van der Waals forces, and red represents repulsive interactions. Calculated with M06/def2‐TZVP.

Analyzing the 3D NCI values (Figure 7) it reveals that blue regions indicate strong interaction energies or a hydrogen bond, green regions correspond to weak van der Waals interactions, and red regions represent repulsive interactions. The linear interactions between ${\mathrm{CO}}_{2}$ and Ti observed in clusters 1, 2, 3, and 4 display blue regions, which indicates a strong interaction between the Ti atom of the cluster and the oxygen atom of ${\mathrm{CO}}_{2}$. In the larger clusters (5, 6, 7, 8, and 9), when the interaction geometry becomes angular, red and green regions also appear. Notably, the orientation between the Ti atom of the cluster and the ${\mathrm{CO}}_{2}$ oxygen atom is consistently marked in blue. However, interactions involving the ${\mathrm{CO}}_{2}$ carbon atom and other cluster atoms tend to be repulsive or weak. This suggests that Ti prefers to interact with the oxygen atom. Interaction regions can also be analyzed using the 2D color map presented in Figure 8.

To compare the 2D maps, we analyzed the map of the isolated cluster (without ${\mathrm{CO}}_{2}$) and the map with ${\mathrm{CO}}_{2}$ present. The graphs corresponding to clusters n = 1, 2, 3, 4, 7, and 9 show a higher incidence of blue regions, as indicated in Figures S1–S10. Cluster 10 exhibits the lowest interaction energy, and this can also be seen in the little difference between the 2D maps of the cluster and the cluster, which aligns with our interaction energy analysis, as seen in Figure 8.

Using the QTAIM analysis is possible to characterize interactions that can be distinguished by the electron density at the Binding Critical Point (BCP) [72, 77]. The results of QTAIM interactions of clusters ${\left({\mathrm{TiO}}_{2}\right)}_{n}$, with *n* = 1–10 with ${\mathrm{CO}}_{2}$ are represented in the Table 1 and Figure S11.

**TABLE 1:** Topological parameters calculated in atomic unit (a.u.) in level theory M06/def2‐TZVP level.

Clusters	BCP	$\rho \left(r\right)$	$\nabla \rho^{2}\left(r\right)$	*V*(*r*)	*G*(*r*)	*H*(*r*)
${\left({\mathrm{TiO}}_{2}\right)}_{1}\cdots {\mathrm{CO}}_{2}$	Ti1–O6	0.033998	0.203755	−0.041316	0.046128	0.004812
${\left({\mathrm{TiO}}_{2}\right)}_{2}\cdots {\mathrm{CO}}_{2}$	Ti1–O9	0.041913	0.261334	0.056637	−0.053961	0.059647
${\left({\mathrm{TiO}}_{2}\right)}_{3}\cdots {\mathrm{CO}}_{2}$	Ti3–O11	0.044158	0.260041	0.108992	−0.055903	0.060457
${\left({\mathrm{TiO}}_{2}\right)}_{4}\cdots {\mathrm{CO}}_{2}$	Ti1–O15	0.040523	0.241961	0.045877	−0.049201	0.054846
${\left({\mathrm{TiO}}_{2}\right)}_{5}\cdots {\mathrm{CO}}_{2}$	Ti2–O18	0.046586	0.252755	0.062328	−0.056932	0.060060
${\left({\mathrm{TiO}}_{2}\right)}_{6}\cdots {\mathrm{CO}}_{2}$	Ti2–O21	0.043653	0.242258	0.040887	−0.052456	0.056510
${\left({\mathrm{TiO}}_{2}\right)}_{7}\cdots {\mathrm{CO}}_{2}$	Ti1–O24	0.042089	0.225186	0.038837	−0.049277	0.052787
${\left({\mathrm{TiO}}_{2}\right)}_{8}\cdots {\mathrm{CO}}_{2}$	Ti5–O26	0.036525	0.195399	0.033145	−0.040910	0.044880
${\left({\mathrm{TiO}}_{2}\right)}_{9}\cdots {\mathrm{CO}}_{2}$	Ti4–O28	0.037585	0.195837	0.026539	−0.042035	0.045497
${\left({\mathrm{TiO}}_{2}\right)}_{10}\cdots {\mathrm{CO}}_{2}$	Ti9–O33	0.042434	0.228927	0.008319	−0.050106	0.053669

In QTAIM, it is possible to evaluate the nature of the bond through the electron density ($\rho \left(r\right)$) and Laplacian electron density ($\nabla^{2}\rho \left(r\right)$), where for values $\nabla^{2}\rho \left(r\right)<0$ a.u. and $\rho \left(r\right)>0.1$ a.u. there are covalent bonds and $\nabla^{2}\rho \left(r\right)>0$ a.u. and $\rho \left(r\right)<0.1$ a.u. non‐covalent interactions [72, 74, 78]. That all interactions of the ${\mathrm{CO}}_{2}$ with clusters are non‐covalent interactions.

Another classification were observed using the parameters $\nabla^{2}\rho \left(r\right)$ and Total electron density $\left(H\left(r\right)\right)$, where in the conditions of $\nabla^{2}\rho \left(r\right)>0$ and $H\left(r\right)<0$ the interaction as partially covalent and $\nabla^{2}\rho \left(r\right)>0$ and $H\left(r\right)>0$ as electrostatic classify [72, 74]. From these results (Table 1 and Figure S11) all interactions between the cluster ${\left({\mathrm{TiO}}_{2}\right)}_{n}$, n=1−10 and ${\mathrm{CO}}_{2}$ are considered electrostatic. These analysis are consistent with the visualized NCI analyses, where the cluster Ti interactions will preferably be to the ${\mathrm{CO}}_{2}$ oxygen atom. The adsorption sites of the most reactive cluster (n=3) and the cluster identified as the most stable in this study, characterized as a possible magic number (n=4), were also investigated regarding the potential activation of other species, such as CH_4_, H_2_O, and H_2_. The observed trend remained consistent, with the interaction predominantly occurring between the oxygen atom and the titanium of the cluster. For all interacting molecules, the n=3 cluster exhibited higher interaction energies compared to the n=4 cluster, which stood out as a possible magic number.

## 4 Conclusion

This study used Density Functional Theory to systematically investigate the stability, reactivity, and ${\mathrm{CO}}_{2}$ interaction with ${\left({\mathrm{TiO}}_{2}\right)}_{n}$ clusters (n = 1–10). The stability function ($\varepsilon^{3}$) identified n = 2, 4, and 8 as “magic number” clusters that exhibit thermodynamic and kinetic stability. These clusters demonstrated suppressed overall electrophilicity (Δω), with n = 2, 4, and 8 decreasing their reactivity value when compared to their neighbors. Conversely, clusters with odd n (3, 5, 7, 9) exhibited reduced stability and increased reactivity. Notably, n = 6 was identified as a reactive outlier, exhibiting high Fukui density $f^{-}\left(r\right)$ and anomalous electronic stabilization (low fractional occupancy number‐weighted density, $N_{\mathrm{FOD}}$).

${\mathrm{CO}}_{2}$ adsorption energies revealed an inverse correlation with cluster stability; unstable clusters (e.g., n = 3, 5, 9) exhibited strong interactions (down to −0.72 eV), while magic number clusters favored weak physisorption (e.g., −0.45 eV for n = 8). QTAIM analysis showed that all interactions of ${\mathrm{CO}}_{2}$ with ${\left({\mathrm{TiO}}_{2}\right)}_{n}$ are electrostatic and Non‐covalent interaction (NCI) analysis confirmed that adsorption occurs preferentially via Ti‐OCO attraction, with repulsive contacts involving the carbon atom of CO_2_ with graphs corresponding to clusters n = 1, 2, 3, 4, 7, and 9 show a higher incidence of blue regions indicating strong attractive interactions and cluster 10 exhibits the lowest interaction energy, and this can also be seen in the little difference between the 2D maps of the cluster. These analyses conclude that the magic number clusters (n = 2, 4, and 8) provide structural integrity for reusable systems, and reactive clusters (e. g., n = 6 and 9) enable strong ${\mathrm{CO}}_{2}$ activation. Future studies should explore photoexcitation dynamics and cluster support synergies to optimize ${\mathrm{CO}}_{2}$ conversion efficiency. A table summarizing all the data presented for each cluster can be found in Table S13 and Figure S12.

To verify whether clusters *n* = 3 and *n* = 4 could activate other molecules, we investigated their interactions with $H_{2}$, $O_{2}$, ${\mathrm{CH}}_{4}$, and $H_{2}O$. The results confirmed consistent stability trends for the clusters, with *n* = 3 remaining the most reactive and *n* = 4 maintaining their higher stability across all cases. The strongest interaction occurs with the water molecule, followed by ${\mathrm{CO}}_{2}$. Water exhibits high interaction energy when binding to the Ti site. In contrast, for the oxygen site, the strongest activation is observed with ${\mathrm{CO}}_{2}$. This suggests that, in photocatalytic interaction, water preferentially occupies the Ti interaction sites, while ${\mathrm{CO}}_{2}$ interacts more effectively with the oxygen sites. These findings further support the previous observations and can be found in detail in the Figure S13 in the SI.

## Conflicts of Interest

The authors declare no conflicts of interest.

## Supporting information


**Data S1:** jcc70232‐sup‐0001‐Supinfo.docx.

## Data Availability

The data that supports the findings of this study are available in the Supporting Information of this article.
