# Supplementary material for: Stability and Reactivity of (TiO2)n, n = 1–10, Clusters and Their Interactions With CO2
Source: J Comput Chem. 2025 Sep 20;46(25):e70232. doi: 10.1002/jcc.70232 (PMC12450047; doi:10.1002/jcc.70232)
Supplement: Supplementary file 1 — Data S1: jcc70232‐sup‐0001‐Supinfo.docx. [file JCC-46-0-s001.docx]

**Supplementary Material: Stability and Reactivity of (TiO_2_)_n_ (n=1–10) Clusters and Their Interactions with CO_2_**

^1^Letícia Carolaine Silva Faria, ^1^Letícia Marques de Souza Vetrano de Queiroz, ^2^Murielly Fernanda Ribeiro Bihain, ^1^Douglas Henrique Pereira, ^1^Leonardo Tsuyoshi Ueno, ^1^Francisco Bolivar Correto Machado*, and ^1^Luiz Fernando de Araujo Ferrão*.

^1^Aeronautics Institute of Technology (ITA), São José dos Campos, SP, Brazil

^2^Federal University of Tocantins (UFT), Gurupi, TO, Brazil.

Summary Table

[Table S 1 - Stability Ranking Function (ε₃). 2](#_Toc205194270)

[Table S 2 - Electrophilicity index (M06/DEF2TZVP). 2](#_Toc205194271)

[Table S 3 – Fukui values n=1. 3](#_Toc205194272)

[Table S 4 – Fukui values n=2. 3](#_Toc205194273)

[Table S 5 – Fukui values n=3 3](#_Toc205194274)

[Table S 6 – Fukui values n=4 4](#_Toc205194275)

[Table S 7– Fukui values n=5 4](#_Toc205194276)

[Table S 8 – Fukui values n=6 5](#_Toc205194277)

[Table S 9 – Fukui values n=7 6](#_Toc205194278)

[Table S 10 – Fukui values n=8. 6](#_Toc205194279)

[Table S 11 – Fukui values n=9. 7](#_Toc205194280)

[Table S 12 – Fukui values n=10. 8](#_Toc205194281)

[Table S 13 Summary of quantitative and qualitative properties for each cluster (n = 1–10), compared to the previous one. 16](#_Toc205194282)

Summary Figure

[Figure S 1- 2D visualization of non-covalent interactions (NCI) between CO₂ and (TiO₂)_1_ clusters at optimal adsorption sites, as well as only (TiO₂)_1_. 10](#_Toc207107234)

[Figure S 2- 2D visualization of non-covalent interactions (NCI) between CO₂ and (TiO₂)_2_ clusters at optimal adsorption sites, as well as only (TiO₂)_2_. 11](#_Toc207107235)

[Figure S 3- 2D visualization of non-covalent interactions (NCI) between CO₂ and (TiO₂)_3_ clusters at optimal adsorption sites, as well as only (TiO₂)_3_. 12](#_Toc207107236)

[Figure S 4- 2D visualization of non-covalent interactions (NCI) between CO₂ and (TiO₂)_4_ clusters at optimal adsorption sites, as well as only (TiO₂)_4_. 13](#_Toc207107237)

[Figure S 5- 2D visualization of non-covalent interactions (NCI) between CO₂ and (TiO₂)_5_ clusters at optimal adsorption sites, as well as only (TiO₂)_5_. 13](#_Toc207107238)

[Figure S 6- 2D visualization of non-covalent interactions (NCI) between CO₂ and (TiO₂)_6_ clusters at optimal adsorption sites, as well as only (TiO₂)_6_. 14](#_Toc207107239)

[Figure S 7- 2D visualization of non-covalent interactions (NCI) between CO₂ and (TiO₂)_7_ clusters at optimal adsorption sites, as well as only (TiO₂)_7_. 14](#_Toc207107240)

[Figure S 8- 2D visualization of non-covalent interactions (NCI) between CO₂ and (TiO₂)_8_ clusters at optimal adsorption sites, as well as only (TiO₂)_8_. 15](#_Toc207107241)

[Figure S 9- 2D visualization of non-covalent interactions (NCI) between CO₂ and (TiO₂)_9_ clusters at optimal adsorption sites, as well as only (TiO₂)_9_. 15](#_Toc207107242)

[Figure S 10- 2D visualization of non-covalent interactions (NCI) between CO₂ and (TiO₂)_10_ clusters at optimal adsorption sites, as well as only (TiO₂)_10_. 15](#_Toc207107243)

[Figure S 11- Molecular table generated by QTAIM with the respective Bond critical points (BCP) highlighted interactions 16](#_Toc207107244)

[Figure S 12 : Summary of the qualitative properties for each cluster (n = 1–10) is compared to the previous analysis, which had a higher value. 17](#_Toc207107245)

[Figure S 13 Interaction energies (in eV) of clusters n = 3 and n= 4 with different molecules (H₂, O₂, CH₄, and H₂O) at the identified Ti and O interaction sites. 17](#_Toc207107246)

Table S 1 - Stability Ranking Function (ε₃).

All calculations were performed using the M06 functional and the def2-TZVP basis set.

| (TiO_2_)_n_ | E _ST_(eV) | IP (eV) | Gibbs atom (E/n) (eV) | ε^3^ (eV) |
| --- | --- | --- | --- | --- |
| n=1 | 2.42 | 9.56 | 3.99 | 92.30 |
| n=2 | 3.95 | 10.21 | 4.98 | 200.94 |
| n=3 | 2.14 | 9.70 | 5.24 | 108.96 |
| n=4 | 3.62 | 9.46 | 5.39 | 184.46 |
| n=5 | 3.24 | 9.27 | 5.47 | 164.42h |
| n=6 | 2.52 | 9.22 | 5.58 | 129.79 |
| n=7 | 2.50 | 9.92 | 5.58 | 138.21 |
| n=8 | 2.68 | 10.20 | 5.66 | 154.82 |
| n=9 | 2.47 | 8.63 | 5.63 | 120.12 |
| n=10 | 3.85 | 9.27 | 5.70 | 203.03 |

Table S 2 - Electrophilicity index (M06/DEF2TZVP).

| (TiO_2_)_n_ | EA (eV) | μ (eV) | η (eV) | ${\Delta\boldsymbol{\omega}}^{\pm}$ |
| --- | --- | --- | --- | --- |
| n=1 | 1.19 | -5.38 | 8.38 | 1.72 |
| n=2 | 1.46 | -5.84 | 8.75 | 1.94 |
| n=3 | 2.66 | -6.18 | 7.04 | 2.71 |
| n=4 | 1.86 | -5.66 | 7.60 | 2.11 |
| n=5 | 2.38 | -5.83 | 6.89 | 2.46 |
| n=6 | 2.95 | -6.09 | 6.27 | 2.95 |
| n=7 | 3.20 | -6.56 | 6.72 | 3.20 |
| n=8 | 2.79 | -6.49 | 7.41 | 2.84 |
| n=9 | 3.04 | -5.83 | 5.59 | 3.04 |
| n=10 | 3.34 | -6.30 | 5.93 | 3.35 |

Calculation of Fukui indices with SCF methods: Kohn-Sham (DFJ-RKS). Functional: MN-15L (except for cluster n=9 and n=10. which was done for M06). Basis: DEF2-TZVP

Table S 3 – Fukui values n=1.

| atom | Charge neutral | Charge cation | Charge anion | F^-^ | F^+^ |
| --- | --- | --- | --- | --- | --- |
| Ti | 1.30485 | 1.50882 | 0.56866 | -0.20397 | -0.73619 |
| O | -0.65234 | -0.25448 | -0.78426 | -0.39786 | -0.13192 |
| O | -0.65251 | -0.25433 | -0.78440 | -0.39818 | -0.13189 |

Table S 4 – Fukui values n=2.

| atom | Charge neutral | Charge cation | Charge anion | F^-^ | F^+^ |
| --- | --- | --- | --- | --- | --- |
| Ti | 1.366 | 1.46365 | 1.03733 | -0.098 | -0.328 |
| Ti | 1.365 | 1.46273 | 1.03769 | -0.098 | -0.327 |
| O | -0.596 | -0.34915 | -0.7097 | -0.246 | -0.114 |
| O | -0.770 | -0.61646 | -0.82845 | -0.154 | -0.058 |
| O | -0.770 | -0.61269 | -0.8283 | -0.157 | -0.058 |
| O | -0.595 | -0.34807 | -0.70858 | -0.247 | -0.114 |

Table S 5 – Fukui values n=3

| atom | Charge neutral | Charge cation | Charge anion | F^-^ | F^+^ |
| --- | --- | --- | --- | --- | --- |
| Ti | 1.415 | 1.4744 | 1.3987 | -0.0594 | -0.0163 |
| Ti | 1.4154 | 1.4746 | 1.3992 | -0.0592 | -0.0162 |
| Ti | 1.3762 | 1.4498 | 0.8603 | -0.0736 | -0.5159 |
| O | -0.7325 | -0.6820 | -0.8287 | -0.0505 | -0.0962 |
| O | -0.588 | -0.3561 | -0.6673 | -0.2319 | -0.0793 |
| O | -0.8095 | -0.5891 | -0.8536 | -0.2204 | -0.0441 |
| O | -0.7562 | -0.7339 | -0.8127 | -0.0223 | -0.0565 |
| O | -0.7325 | -0.6819 | -0.8288 | -0.0506 | -0.0963 |
| O | -0.5877 | -0.3557 | -0.6670 | -0.2320 | -0.0793 |

Table S 6 – Fukui values n=4

| atom | Charge neutral | Charge cation | Charge anion | F^-^ | F^+^ |
| --- | --- | --- | --- | --- | --- |
| Ti | 1.4625 | 1.5151 | 1.32637 | -0.0526 | -0.136 |
| Ti | 1.4626 | 1.5151 | 1.30241 | -0.0525 | -0.160 |
| Ti | 1.4475 | 1.4994 | 1.40926 | -0.0519 | -0.038 |
| Ti | 1.4467 | 1.4984 | 1.4146 | -0.0517 | -0.032 |
| O | -0.8102 | -0.7201 | -0.8846 | -0.0902 | -0.074 |
| O | -0.5536 | -0.3615 | -0.6493 | -0.1921 | -0.096 |
| O | -0.5532 | -0.3608 | -0.6499 | -0.1924 | -0.097 |
| O | -0.8108 | -0.7215 | -0.6499 | -0.0893 | 0.161 |
| O | -0.7431 | -0.6915 | -0.8827 | -0.0516 | -0.140 |
| O | -0.7272 | -0.7308 | -0.8399 | 0.0036 | -0.113 |
| O | -0.8106 | -0.7213 | -0.7761 | -0.0893 | 0.035 |
| O | -0.8104 | -0.7203 | -0.8845 | -0.0901 | -0.074 |

Table S 7– Fukui values n=5

| atom | Charge neutral | Charge cation | Charge anion | F^-^ | F^+^ |
| --- | --- | --- | --- | --- | --- |
| Ti | 1.4621 | 1.5039 | 1.3543 | -0.0417 | -0.10786 |
| Ti | 1.4647 | 1.5059 | 1.3376 | -0.0412 | -0.12713 |
| Ti | 1.4617 | 1.5032 | 1.3499 | -0.0415 | -0.1118 |
| Ti | 1.4271 | 1.4805 | 1.4077 | -0.0534 | -0.0194 |
| Ti | 1.4014 | 1.4509 | 1.3352 | -0.0495 | -0.0662 |
| O | -0.7680 | -0.7031 | -0.8292 | -0.0649 | -0.0612 |
| O | -0.7927 | -0.702 | -0.8528 | -0.0907 | -0.0601 |
| O | -0.5385 | -0.3833 | -0.6146 | -0.1552 | -0.0761 |
| O | -0.7342 | -0.7479 | -0.7312 | 0.0137 | 0.003 |
| O | -0.7932 | -0.7014 | -0.8533 | -0.0918 | -0.0601 |
| O | -0.8061 | -0.7565 | -0.8578 | -0.0496 | -0.0517 |
| O | -0.7681 | -0.7023 | -0.8288 | -0.0658 | -0.0607 |
| O | -0.5351 | -0.3399 | -0.6265 | -0.1952 | -0.0914 |
| O | -0.8061 | -0.7564 | -0.8584 | -0.0497 | -0.0523 |
| O | -0.675 | -0.6514 | -0.7320 | -0.0236 | -0.0570 |

Table S 8 – Fukui values n=6

| atom | Charge neutral | Charge cation | Charge anion | F^-^ | F^+^ |
| --- | --- | --- | --- | --- | --- |
| Ti | 1.4732 | 1.5069 | 1.4014 | -0.0337 | -0.0718 |
| Ti | 1.4615 | 1.4949 | 1.3629 | -0.0334 | -0.0986 |
| Ti | 1.4195 | 1.4755 | 1.4159 | -0.056 | -0.0036 |
| Ti | 1.4186 | 1.475 | 1.415 | -0.0564 | -0.0036 |
| Ti | 1.4731 | 1.507 | 1.4009 | -0.0339 | -0.0722 |
| Ti | 1.4617 | 1.4952 | 1.3626 | -0.0335 | -0.0991 |
| O | -0.7666 | -0.7038 | -0.8177 | -0.0628 | -0.0511 |
| O | -0.7554 | -0.7131 | -0.8168 | -0.0423 | -0.0614 |
| O | -0.7665 | -0.7042 | -0.8172 | -0.0623 | -0.0507 |
| O | -0.5397 | -0.3541 | -0.6183 | -0.1856 | -0.0786 |
| O | -0.7453 | -0.7068 | -0.7903 | -0.0385 | -0.045 |
| O | -0.7551 | -0.7128 | -0.8166 | -0.0423 | -0.0615 |
| O | -0.752 | -0.7219 | -0.8121 | -0.0301 | -0.0601 |
| O | -0.7453 | -0.708 | -0.79 | -0.0373 | -0.0447 |
| O | -0.5392 | -0.3529 | -0.6177 | -0.1863 | -0.0785 |
| O | -0.7518 | -0.7223 | -0.8118 | -0.0295 | -0.06 |
| O | -0.7931 | -0.803 | -0.7863 | 0.0099 | 0.0068 |
| O | -0.7973 | -0.7512 | -0.8633 | -0.0461 | -0.066 |

Table S 9 – Fukui values n=7

| atom | Charge neutral | Charge cation | Charge anion | F^-^ | F^+^ |
| --- | --- | --- | --- | --- | --- |
| Ti | 1.485 | 1.513 | 1.388 | -0.028 | -0.097 |
| Ti | 1.427 | 1.473 | 1.423 | -0.046 | -0.004 |
| Ti | 1.484 | 1.512 | 1.389 | -0.028 | -0.095 |
| Ti | 1.427 | 1.476 | 1.423 | -0.049 | -0.004 |
| Ti | 1.472 | 1.494 | 1.458 | -0.021 | -0.014 |
| Ti | 1.484 | 1.512 | 1.393 | -0.028 | -0.091 |
| Ti | 1.484 | 1.511 | 1.387 | -0.028 | -0.096 |
| O | -0.771 | -0.751 | -0.819 | -0.020 | -0.047 |
| O | -0.536 | -0.363 | -0.604 | -0.173 | -0.068 |
| O | -0.751 | -0.748 | -0.767 | -0.003 | -0.016 |
| O | -0.816 | -0.769 | -0.863 | -0.047 | -0.047 |
| O | -0.772 | -0.751 | -0.819 | -0.021 | -0.048 |
| O | -0.771 | -0.748 | -0.817 | -0.023 | -0.046 |
| O | -0.720 | -0.698 | -0.765 | -0.022 | -0.045 |
| O | -0.816 | -0.770 | -0.868 | -0.046 | -0.052 |
| O | -0.772 | -0.749 | -0.819 | -0.023 | -0.048 |
| O | -0.720 | -0.698 | -0.763 | -0.022 | -0.043 |
| O | -0.766 | -0.662 | -0.794 | -0.104 | -0.028 |
| O | -0.751 | -0.748 | -0.766 | -0.003 | -0.015 |
| O | -0.536 | -0.363 | -0.603 | -0.173 | -0.067 |
| O | -0.766 | -0.662 | -0.795 | -0.104 | -0.029 |

Table S 10 – Fukui values n=8.

| atom | Charge neutral | Charge cation | Charge anion | F^-^ | F^+^ |
| --- | --- | --- | --- | --- | --- |
| Ti | 1.379 | 1.37758 | 1.4289 | 0.001 | 0.050 |
| Ti | 1.3798 | 1.37758 | 1.4289 | 0.002 | 0.049 |
| Ti | 1.4338 | 1.37499 | 1.4583 | 0.059 | 0.025 |
| Ti | 1.4337 | 1.37499 | 1.4583 | 0.059 | 0.025 |
| Ti | 1.4337 | 1.37499 | 1.4583 | 0.059 | 0.025 |
| Ti | 1.4338 | 1.37499 | 1.4583 | 0.059 | 0.025 |
| Ti | 1.4157 | 1.35389 | 1.448 | 0.062 | 0.032 |
| Ti | 1.4157 | 1.35389 | 1.448 | 0.062 | 0.032 |
| O | -0.5085 | -0.5794 | -0.3562 | 0.071 | 0.152 |
| O | -0.5085 | -0.5794 | -0.3562 | 0.071 | 0.152 |
| O | -0.7324 | -0.7776 | -0.6998 | 0.045 | 0.033 |
| O | -0.7325 | -0.7776 | -0.6998 | 0.045 | 0.033 |
| O | -0.7325 | -0.7776 | -0.6998 | 0.045 | 0.033 |
| O | -0.7325 | -0.7776 | -0.6998 | 0.045 | 0.033 |
| O | -0.7599 | -0.809 | -0.7136 | 0.049 | 0.046 |
| O | -0.76 | -0.809 | -0.7136 | 0.049 | 0.046 |
| O | -0.7488 | -0.784 | -0.7136 | 0.035 | 0.035 |
| O | -0.7488 | -0.784 | -0.7136 | 0.035 | 0.035 |
| O | -0.7416 | -0.771 | -0.7172 | 0.029 | 0.024 |
| O | -0.7416 | -0.771 | -0.7172 | 0.029 | 0.024 |
| O | -0.679 | -0.672 | -0.679 | -0.007 | 0.000 |
| O | -0.679 | -0.672 | -0.679 | -0.007 | 0.000 |
| O | -0.759 | -0.8092 | -0.7136 | 0.050 | 0.045 |
| O | -0.76 | -0.8092 | -0.7136 | 0.049 | 0.046 |

Table S 11 – Fukui values n=9.

| atom | Charge neutral | Charge cation | Charge anion | F^-^ | F^+^ |
| --- | --- | --- | --- | --- | --- |
| Ti | 1.416 | 1.365 | 1.4506 | 0.051 | 0.0346 |
| Ti | 1.425 | 1.366 | 1.4515 | 0.059 | 0.0266 |
| Ti | 1.419 | 1.365 | 1.4349 | 0.054 | 0.0156 |
| Ti | 1.403 | 1.364 | 1.4379 | 0.039 | 0.0350 |
| Ti | 1.415 | 1.406 | 1.4286 | 0.009 | 0.0135 |
| Ti | 1.409 | 1.389 | 1.4434 | 0.020 | 0.0343 |
| Ti | 1.468 | 1.380 | 1.4809 | 0.088 | 0.0133 |
| Ti | 1.440 | 1.368 | 1.4621 | 0.071 | 0.0226 |
| Ti | 1.354 | 1.350 | 1.3833 | 0.004 | 0.0293 |
| O | -0.647 | -0.675 | -0.5973 | 0.028 | 0.0499 |
| O | -0.723 | -0.752 | -0.6457 | 0.029 | 0.0770 |
| O | -0.717 | -0.770 | -0.6744 | 0.052 | 0.0428 |
| O | -0.702 | -0.737 | -0.6550 | 0.035 | 0.0468 |
| O | -0.492 | -0.533 | -0.3732 | 0.042 | 0.1186 |
| O | -0.749 | -0.799 | -0.7177 | 0.050 | 0.0312 |
| O | -0.701 | -0.740 | -0.6572 | 0.039 | 0.0438 |
| O | -0.699 | -0.757 | -0.6635 | 0.058 | 0.0354 |
| O | -0.753 | -0.798 | -0.6983 | 0.045 | 0.0550 |
| O | -0.772 | -0.828 | -0.7306 | 0.056 | 0.0409 |
| O | -0.695 | -0.740 | -0.6446 | 0.045 | 0.0507 |
| O | -0.736 | -0.745 | -0.7294 | 0.009 | 0.0065 |
| O | -0.742 | -0.738 | -0.7491 | -0.004 | -0.0073 |
| O | -0.751 | -0.741 | -0.7514 | -0.010 | -0.0005 |
| O | -0.683 | -0.703 | -0.6296 | 0.020 | 0.0531 |
| O | -0.743 | -0.750 | -0.7376 | 0.007 | 0.0050 |
| O | -0.695 | -0.739 | -0.6091 | 0.044 | 0.0860 |
| O | -0.761 | -0.810 | -0.7092 | 0.050 | 0.0513 |

Table S 12 – Fukui values n=10.

| atom | Charge neutral | Charge cation | Charge anion | F^-^ | F^+^ |
| --- | --- | --- | --- | --- | --- |
| Ti | 1.392 | 1.346 | 1.4261 | 0.046 | 0.034 |
| Ti | 1.397 | 1.386 | 1.4088 | 0.011 | 0.012 |
| Ti | 1.388 | 1.378 | 1.3994 | 0.009 | 0.012 |
| Ti | 1.207 | 1.091 | 1.2412 | 0.116 | 0.034 |
| Ti | 1.383 | 1.379 | 1.383 | 0.004 | 0.000 |
| Ti | 1.415 | 1.403 | 1.4227 | 0.013 | 0.008 |
| Ti | 1.417 | 1.389 | 1.4393 | 0.028 | 0.023 |
| Ti | 1.379 | 1.349 | 1.405 | 0.030 | 0.026 |
| Ti | 1.444 | 1.422 | 1.462 | 0.022 | 0.018 |
| Ti | 0.829 | 0.792 | 0.889 | 0.036 | 0.060 |
| O | -0.692 | -0.713 | -0.674 | 0.021 | 0.018 |
| O | -0.756 | -0.788 | -0.722 | 0.032 | 0.034 |
| O | -0.731 | -0.759 | -0.704 | 0.028 | 0.027 |
| O | -0.749 | -0.779 | -0.721 | 0.030 | 0.028 |
| O | -0.741 | -0.817 | -0.681 | 0.076 | 0.060 |
| O | -0.761 | -0.753 | -0.773 | -0.008 | -0.012 |
| O | -0.710 | -0.710 | -0.715 | 0.000 | -0.005 |
| O | -0.696 | -0.724 | -0.664 | 0.028 | 0.032 |
| O | 0.094 | 0.081 | 0.108 | 0.013 | 0.014 |
| O | -0.723 | -0.758 | -0.685 | 0.035 | 0.038 |
| O | -0.744 | -0.747 | -0.738 | 0.003 | 0.006 |
| O | -0.625 | -0.652 | -0.59 | 0.027 | 0.035 |
| O | -0.702 | -0.738 | -0.675 | 0.036 | 0.027 |
| O | -0.695 | -0.726 | -0.71 | 0.031 | -0.015 |
| O | -0.727 | -0.764 | -0.686 | 0.037 | 0.041 |
| O | -0.694 | -0.734 | -0.661 | 0.040 | 0.033 |
| O | -0.511 | -0.560 | -0.454 | 0.049 | 0.057 |
| O | -0.690 | -0.794 | -0.442 | 0.104 | 0.248 |
| O | -0.729 | -0.763 | -0.682 | 0.034 | 0.047 |
| O | -0.658 | -0.735 | -0.604 | 0.077 | 0.054 |

Figure S 1- 2D visualization of non-covalent interactions (NCI) between CO₂ and (TiO₂)_1_ clusters at optimal adsorption sites, as well as only (TiO₂)_1_.


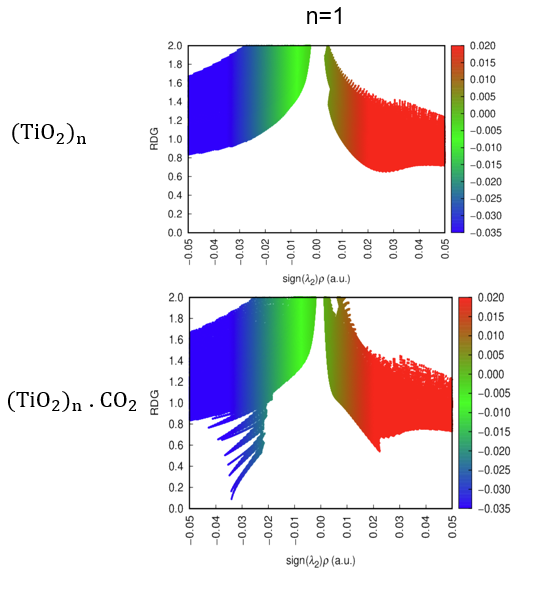


Figure S 2- 2D visualization of non-covalent interactions (NCI) between CO₂ and (TiO₂)_2_ clusters at optimal adsorption sites, as well as only (TiO₂)_2_.


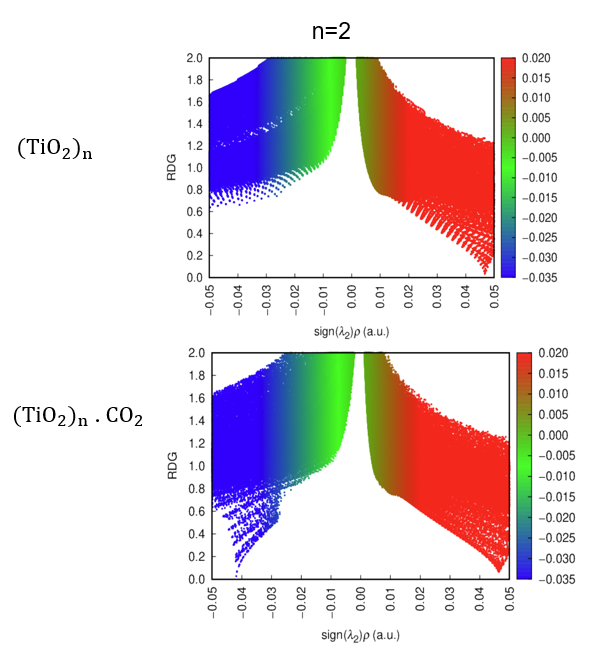


Figure S 3- 2D visualization of non-covalent interactions (NCI) between CO₂ and (TiO₂)_3_ clusters at optimal adsorption sites, as well as only (TiO₂)_3_.


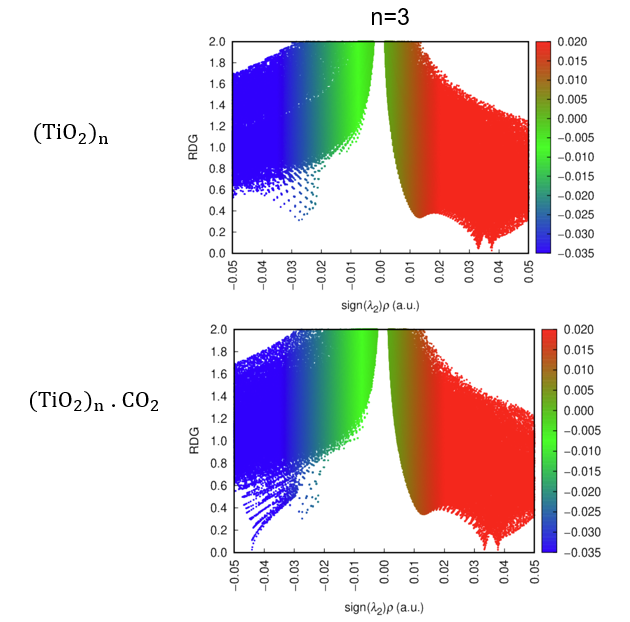


Figure S 4- 2D visualization of non-covalent interactions (NCI) between CO₂ and (TiO₂)_4_ clusters at optimal adsorption sites, as well as only (TiO₂)_4_.


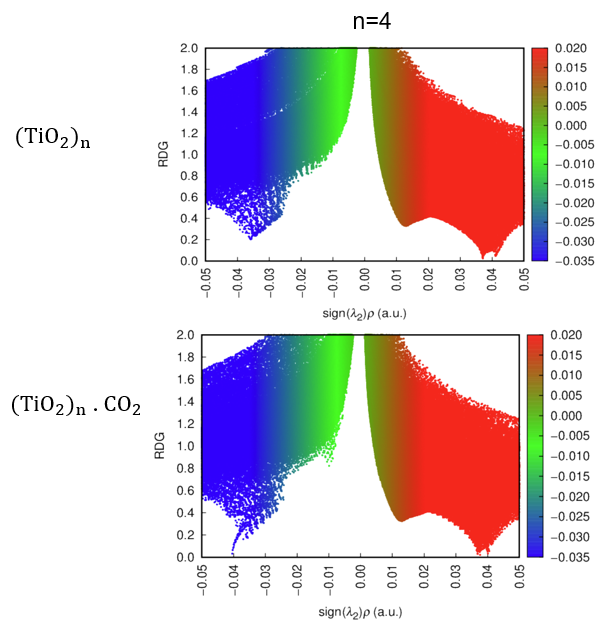


Figure S 5- 2D visualization of non-covalent interactions (NCI) between CO₂ and (TiO₂)_5_ clusters at optimal adsorption sites, as well as only (TiO₂)_5_.


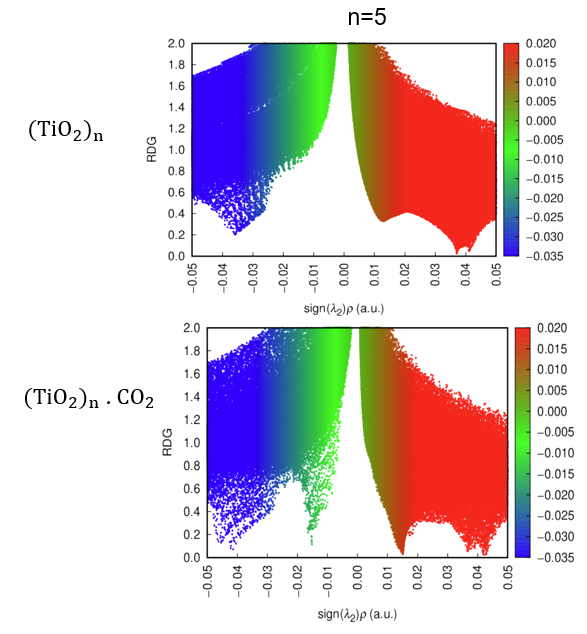


Figure S 6- 2D visualization of non-covalent interactions (NCI) between CO₂ and (TiO₂)_6_ clusters at optimal adsorption sites, as well as only (TiO₂)_6_.


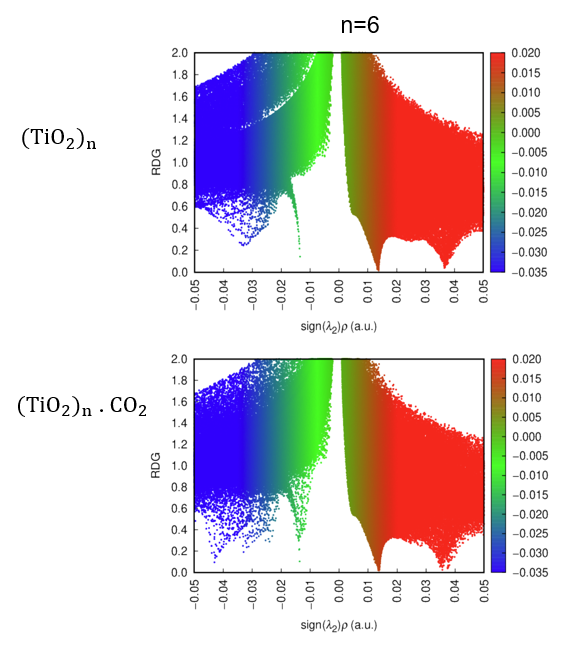


Figure S 7- 2D visualization of non-covalent interactions (NCI) between CO₂ and (TiO₂)_7_ clusters at optimal adsorption sites, as well as only (TiO₂)_7_.


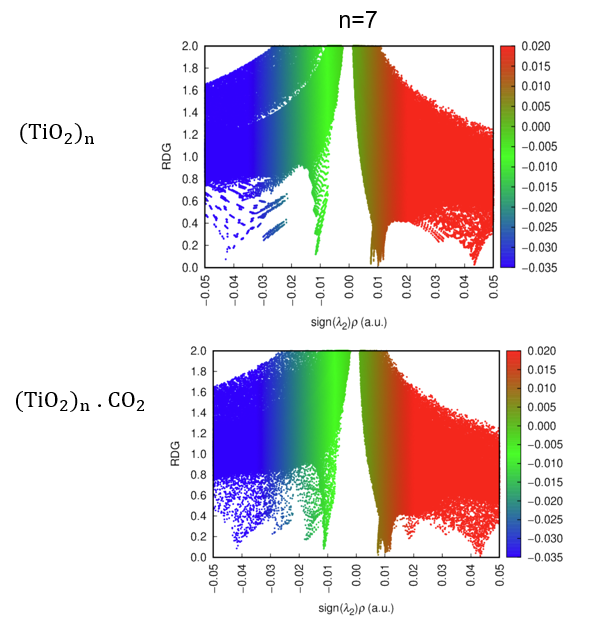


Figure S 8- 2D visualization of non-covalent interactions (NCI) between CO₂ and (TiO₂)_8_ clusters at optimal adsorption sites, as well as only (TiO₂)_8_.


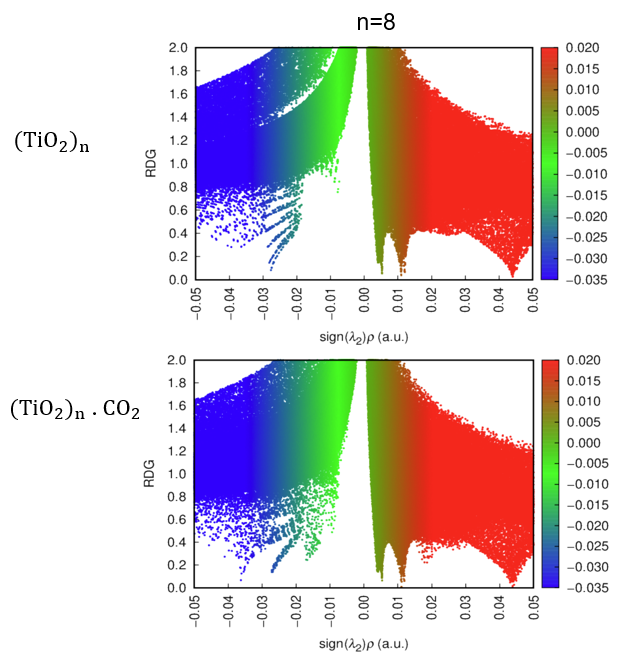


Figure S 9- 2D visualization of non-covalent interactions (NCI) between CO₂ and (TiO₂)_9_ clusters at optimal adsorption sites, as well as only (TiO₂)_9_.


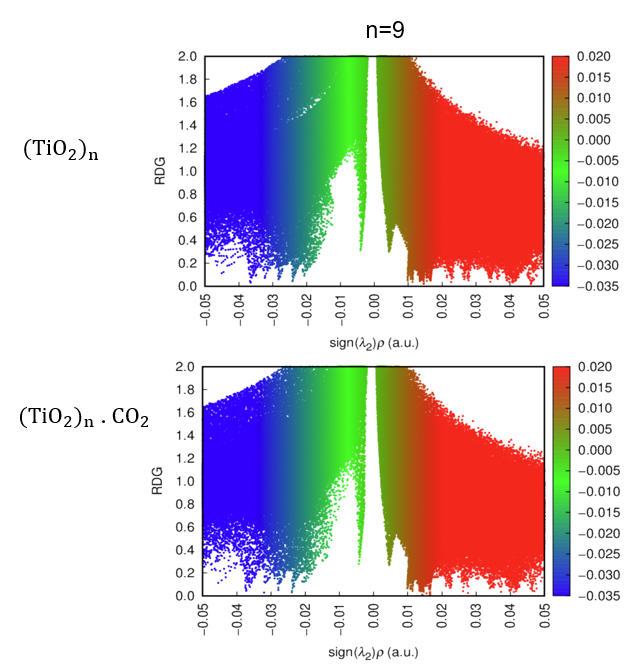


Figure S 10- 2D visualization of non-covalent interactions (NCI) between CO₂ and (TiO₂)_10_ clusters at optimal adsorption sites, as well as only (TiO₂)_10_.


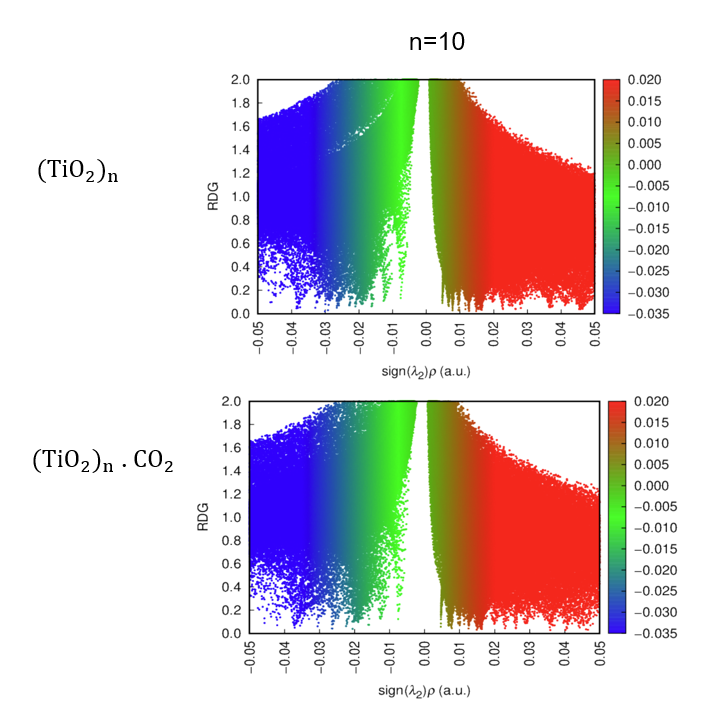


Figure S 11- Molecular table generated by QTAIM with the respective Bond critical points (BCP) highlighted interactions


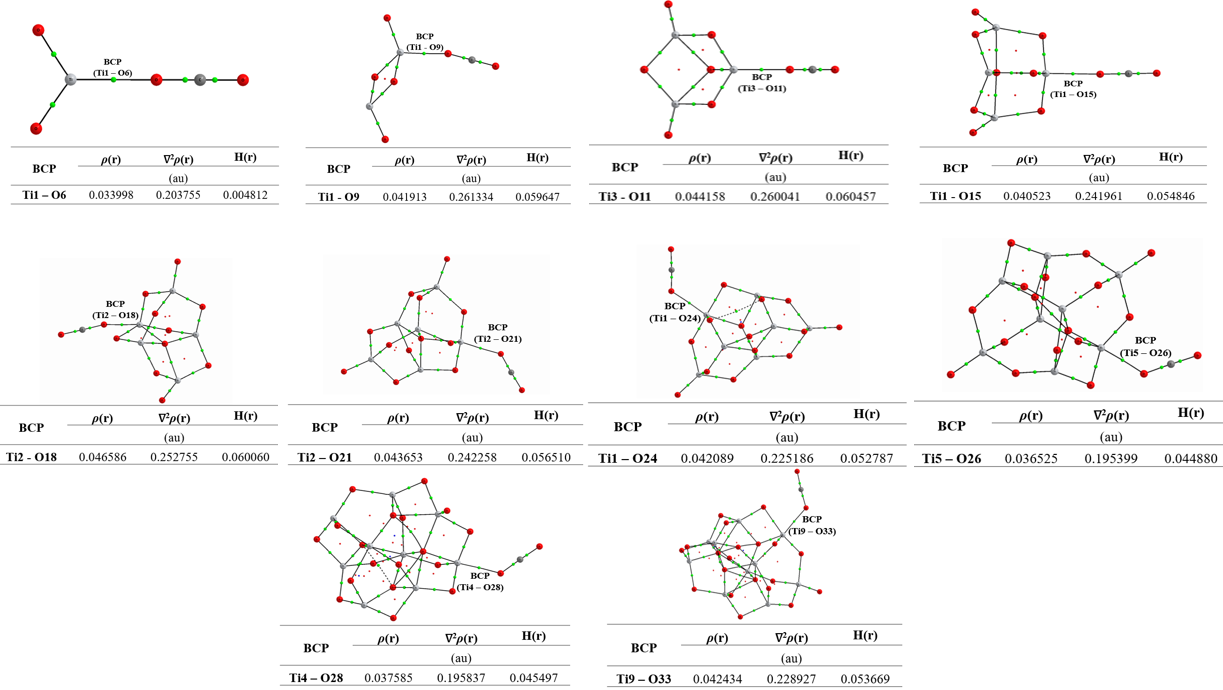


| **n** | $\varepsilon^{3}$ | 𝜟ω^±^ | N_FOD_ | E interaction CO_2_ (eV) |
| --- | --- | --- | --- | --- |
| **1** | 92.30 | 1.72 | 0.277 | -0.50 |
| **2** | 200.94 | 1.94 | 0.152 | -0.63 |
| **3** | 108.96 | 2.71 | 0.156 | -0.72 |
| **4** | 184.46 | 2.11 | 0.138 | -0.54 |
| **5** | 164.42 | 2.46 | 0.130 | -0.66 |
| **6** | 129.79 | 2.95 | 0.129 | -0.58 |
| **7** | 138.21 | 3.20 | 0.143 | -0.5 |
| **8** | 154.8 | 2.84 | 0.119 | -0.45 |
| **9** | 120.12 | 3.04 | 0.133 | -0.64 |
| 10 | 203.0 | 3.3 | 0.125 | -0.35 |

Table S 13- Summary of quantitative and qualitative properties for each cluster (n = 1–10), compared to the previous one.

Figure S 12 : Summary of the qualitative properties for each cluster (n = 1–10) is compared to the previous analysis, which had a higher value.


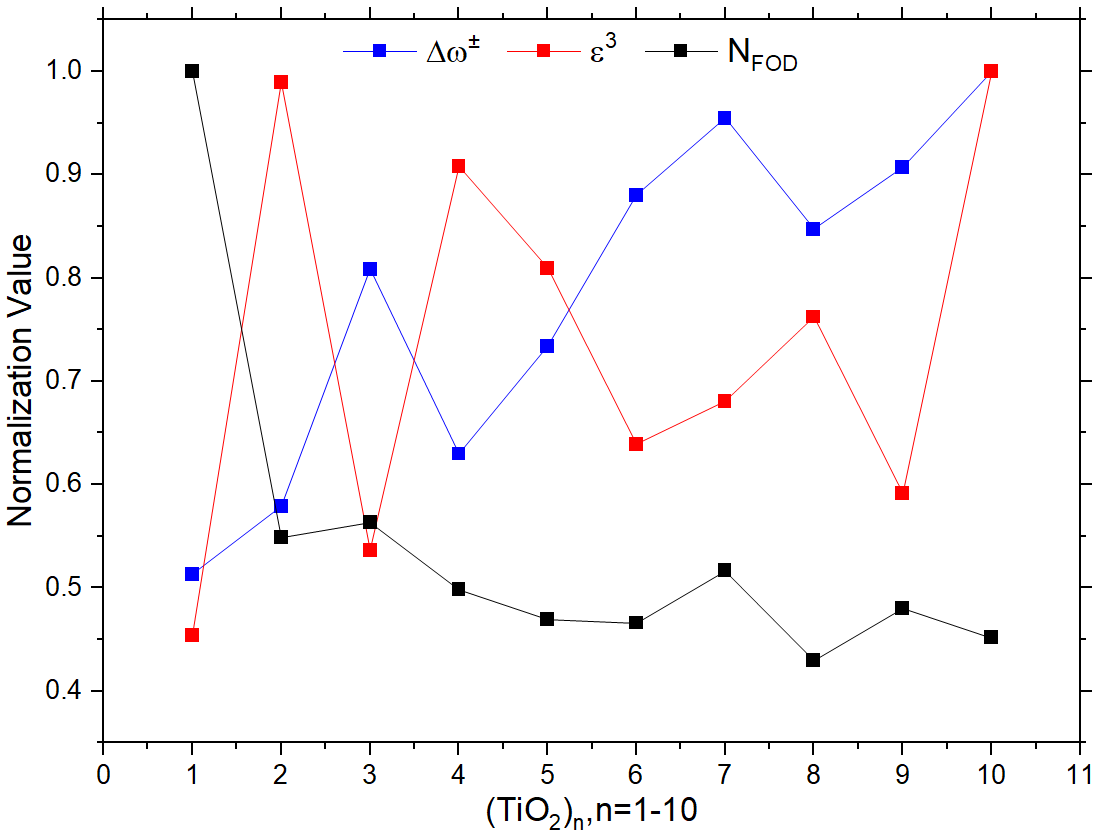


Figure S 13 Interaction energies (in eV) of clusters n = 3 and n= 4 with different molecules (H₂, O₂, CH₄, and H₂O) at the identified Ti and O interaction sites.


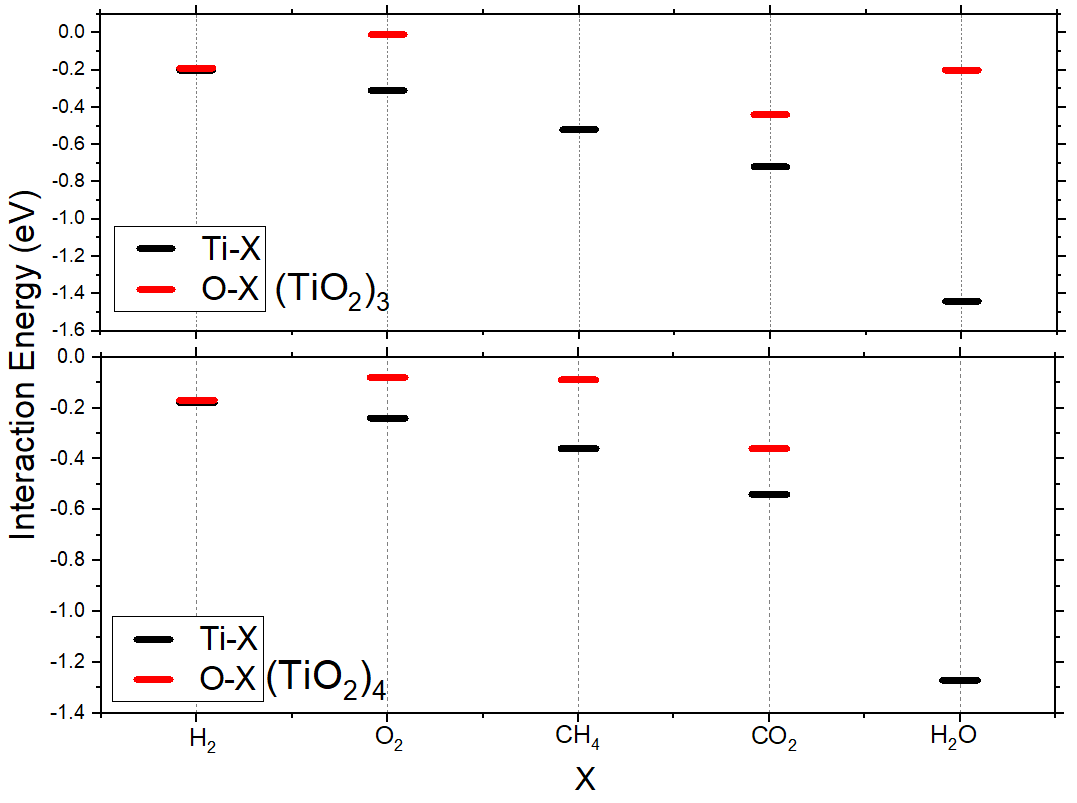


Geometry of clusters.

(TiO2)1 - OPT= M06/DEF2TZVP


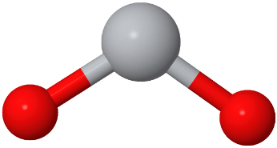


Ti  0.000000 0.000000 0.384337

O  0.000000 1.354234 -0.528463

O  0.000000 -1.354234 -0.528463

(TiO2)2 - OPT= M06/DEF2TZVP


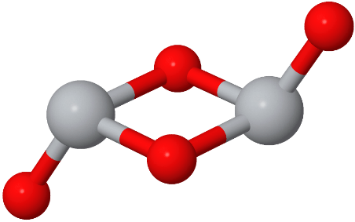


Ti 0.000000 1.347773 0.000000

Ti 0.000000 -1.347773 0.000000

O -1.356537 2.227067 0.000000

O 0.000000 0.000000 1.250245

O 0.000000 0.000000 -1.250245

O 1.356537 -2.227067 0.000000

(TiO2)3 - OPT= M06/DEF2TZVP


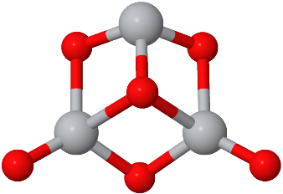


Ti -1.421296 -0.613898 -0.132440

Ti 1.421310 -0.613874 -0.132433

Ti -0.000021 1.756968 0.245786

O -1.407857 1.326308 -0.651949

O 2.694899 -1.358448 0.520799

O 0.000016 -1.583152 -0.816293

O 0.000006 0.192187 1.131083

O 1.407810 1.326322 -0.651964

O -2.694855 -1.358505 0.520812

(TiO2)4 - OPT= M06/DEF2TZVP


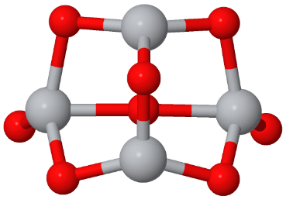


Ti -0.827989 -0.290087 1.406717

Ti -0.827989 -0.290087 -1.406717

Ti 1.242598 -1.695275 0.000000

Ti -0.089942 2.100287 0.000000

O -0.827989 1.476312 1.690229

O 1.082552 3.193103 0.000000

O 2.840104 -1.825499 0.000000

O 0.281359 -1.664349 1.692338

O -1.926142 -0.680583 0.000000

O 0.480883 0.170750 0.000000

O -0.827989 1.476312 -1.690229

O 0.281359 -1.664349 -1.692338

(TiO2)5 - OPT= M06/DEF2TZVP


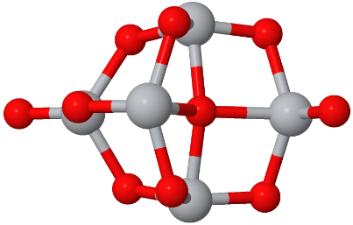


Ti 0.274302 0.120973 1.917587

Ti -1.784286 0.580209 0.000000

Ti 0.274302 0.120973 -1.917587

Ti -0.523126 -2.280228 0.000000

Ti 1.797766 1.692614 0.000000

O 0.274302 -1.613471 -1.653067

O 1.823832 0.999280 -1.798226

O 1.282064 3.210681 0.000000

O 0.173319 0.462680 0.000000

O 1.823832 0.999280 1.798226

O -1.391825 0.925418 -1.790946

O 0.274302 -1.613471 1.653067

O -0.866203 -3.844426 0.000000

O -1.391825 0.925418 1.790946

O -2.108934 -1.096375 0.000000

(TiO2)6 - OPT= M06/DEF2TZVP

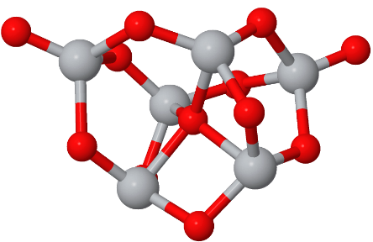


Ti 0.224592 -0.631200 -1.762938

Ti -1.243154 1.783127 0.725245

Ti 2.613353 -0.919005 0.353581

Ti -2.613353 -0.919004 -0.353582

Ti -0.224592 -0.631202 1.762939

Ti 1.243155 1.783128 -0.725244

O 1.435329 -1.197007 1.847788

O 0.858953 1.031033 -2.309601

O -1.435330 -1.197006 -1.847789

O 4.071424 -1.570437 0.477216

O -1.522057 -1.598475 1.122477

O -0.858953 1.031031 2.309603

O 2.607421 1.043195 0.047672

O 1.522057 -1.598475 -1.122480

O -4.071425 -1.570435 -0.477218

O -2.607420 1.043196 -0.047671

O 0.000001 0.370744 0.000000

O 0.000001 2.931566 0.000001

(TiO2)7 - OPT= M06/DEF2TZVP


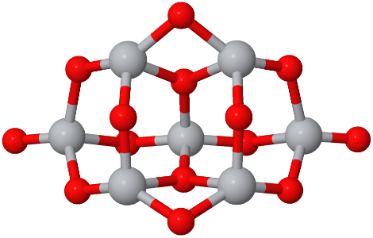


Ti -1.392288 -1.092840 1.621208

Ti -3.117129 1.132343 0.000353

Ti 1.392280 -1.092978 -1.621119

Ti 3.117143 1.132333 -0.000441

Ti 0.000004 1.591602 -0.000077

Ti 1.392821 -1.092440 1.622147

Ti -1.392830 -1.092580 -1.622057

O -2.737010 0.032056 -1.596804

O 4.553475 1.839129 -0.000742

O 0.000248 0.195438 1.281229

O -0.000169 -1.826736 2.629878

O 2.736607 0.031456 -1.597115

O 2.737029 0.032158 1.596789

O -1.454158 -1.897840 -0.000577

O 0.000159 -1.826964 -2.629723

O -2.736593 0.031615 1.597129

O 1.454103 -1.897844 0.000736

O -1.587627 2.334056 0.000256

O -0.000248 0.195331 -1.281263

O -4.553460 1.839138 0.000603

O 1.587640 2.334046 -0.000433

(TiO2)8 - OPT= M06/DEF2TZVP


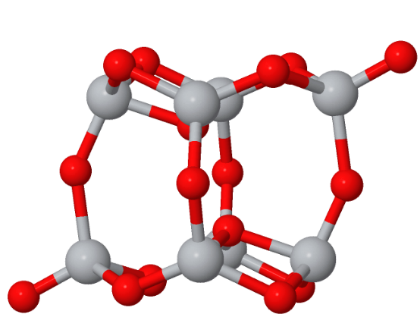


Ti 3.226655 0.143945 0.000005

Ti -3.226651 -0.143981 0.000005

Ti -0.944931 1.331440 1.791512

Ti 0.944928 -1.331411 -1.791501

Ti -0.944938 1.331437 -1.791511

Ti 0.944921 -1.331412 1.791505

Ti -0.736421 -2.695862 -0.000003

Ti 0.736419 2.695858 -0.000006

O 4.812053 0.362279 0.000013

O -4.812054 -0.362279 0.000012

O 2.615325 -0.930188 -1.511374

O -2.615307 0.930070 1.511428

O 2.615317 -0.930174 1.511388

O -2.615314 0.930074 -1.511419

O 0.083685 -2.963635 -1.624775

O -0.083682 2.963664 1.624762

O -2.268783 -1.858770 -0.000036

O 2.268756 1.858709 -0.000048

O 0.000071 0.000071 2.589892

O 0.000074 0.000071 -2.589887

O 0.367184 -1.134427 0.000002

O -0.367233 1.134455 0.000000

O -0.083708 2.963671 -1.624757

O 0.083665 -2.963629 1.624782

(TiO2)9 - OPT= M06/DEF2TZVP


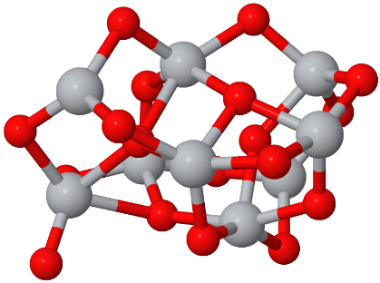


Ti 1.677080 -2.545814 -0.520752

Ti 2.499195 1.962503 -0.434480

Ti -1.019673 -2.164508 -0.849136

Ti -2.867624 -1.094003 1.085109

Ti -0.221736 -0.207108 1.723664

Ti -0.420061 2.701764 0.284616

Ti 0.712865 0.202414 -1.740205

Ti 2.713871 -0.314900 1.300547

Ti -2.646381 1.308582 -0.597949

O 1.918083 -1.335462 -1.727474

O -3.840828 0.271212 0.634668

O 0.236739 -3.477981 -1.207746

O -1.662189 -0.934280 2.518169

O -3.357152 1.299382 -2.029700

O 1.439523 3.270038 0.070420

O 1.460862 -0.338099 2.571017

O 2.051276 1.435656 -2.123131

O -2.621232 -2.559752 -0.042347

O 3.680460 1.168642 0.748923

O -0.616971 -0.981451 -2.208458

O -0.552801 1.563434 -1.155703

O 1.397665 0.467941 0.101798

O -1.500239 -0.339940 0.100732

O -0.464190 1.594775 1.718391

O 0.326358 -1.820642 0.531637

O -2.106606 3.094506 0.093898

O 3.035521 -1.962534 0.713515

TiO2)10 - OPT= M06/DEF2TZVP


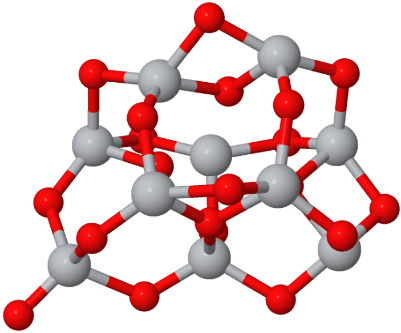


Ti 2.379032 -2.131756 -1.054517

Ti -0.474425 -1.275947 -1.396319

Ti 1.214284 -1.672934 1.605005

Ti 2.136625 1.592349 1.597446

Ti -3.621578 -1.010476 -0.538036

Ti -0.593319 1.853060 1.480970

Ti -2.329482 2.037092 -0.750388

Ti 0.287745 1.529670 -1.685562

Ti -1.493277 -1.313508 2.044451

Ti 3.089383 0.580928 -1.105587

O -1.271894 0.428711 2.401776

O -3.674507 0.922120 -0.870629

O -3.034784 -1.548126 1.263535

O 2.334575 -2.780816 0.656382

O 3.740546 -1.052040 -1.687664

O 1.665133 -0.511213 -0.483771

O -0.371479 -1.536354 0.570623

O -2.132830 -1.704635 -1.585135

O 3.587574 1.445603 0.422103

O 0.846178 -2.542774 -1.871654

O -1.047199 0.774258 -0.329675

O -0.106446 0.058019 -2.601576

O 1.937090 -0.079793 2.191663

O 0.947672 1.892044 0.231815

O 0.047579 -2.182198 2.852267

O -1.939872 2.872307 0.858358

O -5.008893 -1.704094 -0.931553

O 2.134127 1.638031 -2.159960

O -1.094060 2.644772 -2.021872

O 0.805275 2.447867 2.551940

Geometry of clusters and CO_2_.

**(TiO2)1CO2 - (Ti1) - OPT= M06/DEF2TZVP**


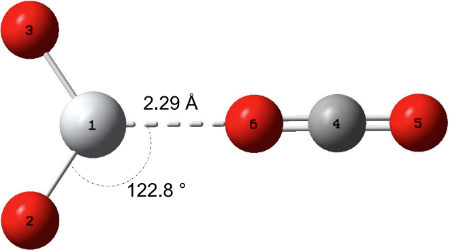


Ti 0.000000 0.000000 1.024938

O 0.000000 1.379589 1.915509

O 0.000000 -1.379589 1.915509

C 0.000000 0.000000 -2.425789

O 0.000000 0.000000 -3.567299

O 0.000000 0.000000 -1.262959

**(TiO2)2CO2 - (Ti1) - OPT= M06/DEF2TZVP**


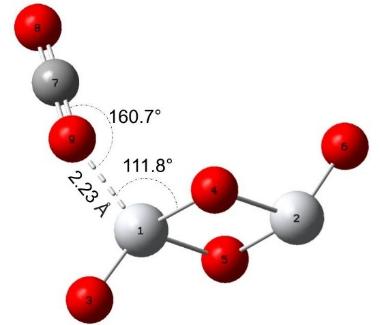


Ti -0.232454 0.976273 -0.007211

Ti 2.016072 -0.511808 0.012297

O -0.326680 2.595446 0.025088

O 0.940070 0.267672 -1.250845

O 0.884436 0.217320 1.257247

O 1.983613 -2.132493 -0.020525

C -3.055664 -0.813975 -0.008463

O -3.922445 -1.555649 0.011288

O -2.172196 -0.059092 -0.029892

**(TiO2)3CO2 - (Ti3) - OPT= M06/DEF2TZVP**


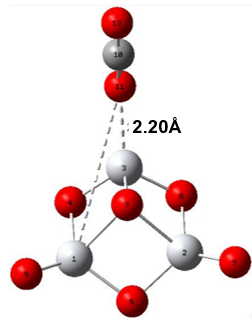


Ti 1.42753 1.42650 0.01309

Ti 1.44964 -1.41416 0.00988

Ti -0.93758 -0.01261 0.31416

O -0.28443 1.41476 1.04900

O 1.99716 -2.68349 -0.82835

O 2.55722 0.01451 0.41464

O 0.32189 -0.00090 -0.97995

O -0.26235 -1.43189 1.04500

O 1.95613 2.70665 -0.82095

C -4.23501 -0.00671 -0.29447

O -5.36008 0.00186 -0.46821

O -3.08317 -0.01572 -0.11744

**(TiO2)4CO2 - (Ti1) - OPT= M06/DEF2TZVP**


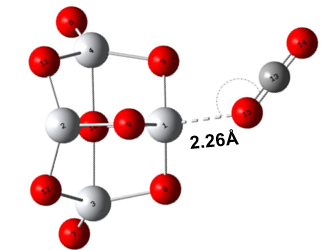


Ti 1.05354 -0.02414 -0.10537

Ti -1.26957 0.01634 1.50648

Ti -1.03924 -1.98514 -0.51305

Ti -0.97138 2.00703 -0.51441

O 0.91968 1.64378 -0.75749

O -1.81299 2.69146 -1.69925

O -1.90363 -2.63985 -1.69812

O 0.86248 -1.68589 -0.75741

O 0.55054 -0.01511 1.65391

O -0.95366 0.01044 -0.42822

O -1.80818 1.70086 1.21578

O -1.86581 -1.64883 1.21672

C 4.41946 -0.03380 0.07915

O 5.56065 -0.01307 0.04497

O 3.25962 -0.05717 0.12219

**(TiO2)5CO2 - (Ti2) - OPT= M06/DEF2TZVP**


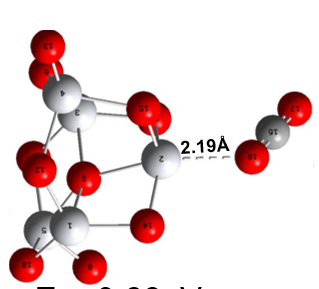


Ti 1.455957 -0.004355 -1.608924

Ti -1.249156 0.194521 -0.708900

Ti -0.072311 -0.312837 1.865292

Ti 0.736519 2.365780 0.372167

Ti 1.496673 -2.193121 0.301124

O 0.573165 1.317758 2.002267

O 1.024866 -1.707075 2.103905

O 0.581605 -3.409745 -0.209199

O 0.483148 -0.460698 0.008033

O 2.466186 -1.412997 -1.172708

O -1.714129 -0.415844 1.038584

O 1.892130 1.577860 -0.985773

O 0.924746 3.942212 0.595465

O -0.272435 -0.108709 -2.266511

O -1.000568 1.863586 -0.435435

C -4.166911 -0.485021 -0.446101

O -4.993201 -0.792294 0.273880

O -3.351456 -0.167756 -1.225019

**(TiO2)6CO2 - (Ti2) - OPT= M06/DEF2TZVP**


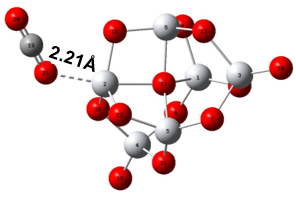


Ti -1.421881 0.758617 -1.522592

Ti 1.877777 -0.237635 0.176459

Ti -2.791509 -1.182840 0.624000

Ti 0.631241 2.748026 -0.147744

Ti -0.347560 0.442848 1.814452

Ti -0.210985 -1.745750 -1.185607

O -1.858648 -0.401884 2.113710

O -0.819565 -0.694405 -2.514325

O -0.701396 2.361882 -1.477124

O -4.211539 -1.844688 0.963292

O -0.264928 2.152502 1.483752

O 1.356887 -0.300222 1.894607

O -1.489735 -2.418532 -0.221638

O -2.849536 0.327354 -0.620130

O 1.167652 4.259072 -0.118759

O 2.053970 1.388642 -0.395800

O -0.187329 -0.058584 -0.132537

O 1.593325 -1.771332 -0.839503

C 4.516141 -1.887127 0.184905

O 5.072727 -2.851726 -0.058326

O 3.974032 -0.886712 0.446939

**(TiO2)7CO2 - (Ti1) - OPT= M06/DEF2TZVP**


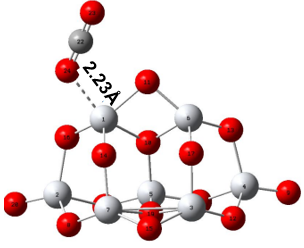


Ti -1.816121 0.922087 -0.400155

Ti -2.190411 -2.334431 -0.742549

Ti 1.720877 -0.010712 1.931835

Ti 3.485134 0.245549 -0.788676

Ti 0.755090 -1.303612 -1.105131

Ti 0.789878 2.005321 -0.413616

Ti -0.825811 -1.124790 1.928333

O -1.729836 -2.423830 1.175293

O 5.003215 0.368366 -1.288347

O -0.035536 0.409744 -1.070295

O -0.934869 2.622611 -0.633259

O 3.267882 -0.188907 1.130603

O 2.366947 1.816532 -1.174663

O -1.596300 0.407543 1.314734

O 0.503132 -0.735567 3.168545

O -2.604815 -0.449353 -1.144275

O 1.076058 1.560868 1.330930

O -0.448117 -2.487656 -1.583804

O 0.719580 -1.195483 0.788535

O -3.302417 -3.384028 -1.223572

O 2.428688 -1.170086 -1.606950

C -3.615079 3.375097 -0.115123

O -3.650879 4.512880 -0.059178

O -3.627673 2.206661 -0.165571

**(TiO2)8CO2 - (Ti5) - OPT= M06/DEF2TZVP**


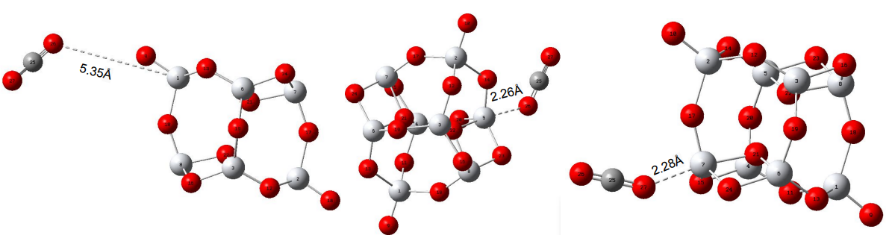


Ti -3.072422 -1.467771 -0.442100

Ti 2.397795 1.767745 0.669433

Ti 0.125854 0.347727 2.468410

Ti -0.766294 -0.068086 -2.240914

Ti 1.683140 -1.272885 -0.386112

Ti -2.288178 1.510772 0.596037

Ti -0.473382 2.553695 -1.280925

Ti -0.190756 -2.250824 1.501999

O -4.371580 -2.385333 -0.628298

O 3.707353 2.675204 0.841641

O -2.211688 -1.030024 -2.141404

O 1.563844 1.329690 2.373387

O -3.496774 0.302337 0.268673

O 2.831713 0.007216 -0.038022

O -0.559386 1.657045 -2.885249

O -0.089782 -1.370697 3.117089

O 1.084949 2.642191 -0.501080

O -1.755467 -2.326001 0.718809

O -1.414444 1.273220 2.167378

O 0.764963 -0.998165 -1.927294

O -0.980526 0.837544 -0.597426

O 0.358128 -0.555922 0.830184

O 1.263823 -2.826329 0.553484

O -1.938191 3.091379 -0.309778

C 4.496143 -1.950625 -1.550448

O 3.408654 -2.353206 -1.421083

O 5.568971 -1.588207 -1.694203

**(TiO2)9CO2 - (Ti4) - OPT= M06/DEF2TZVP**


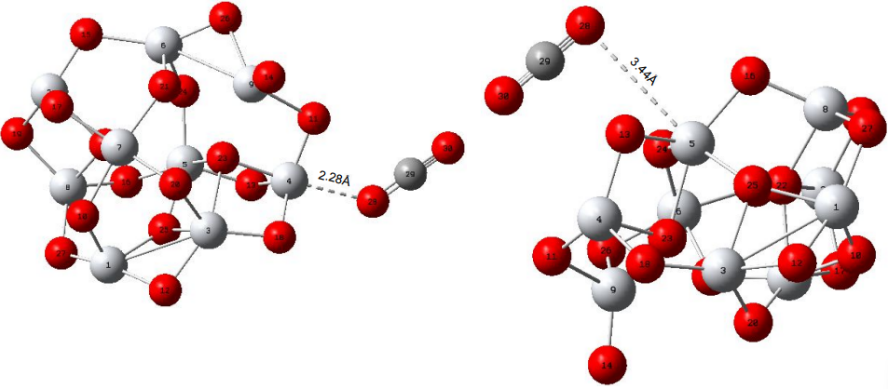


Ti -1.703029 -2.729496 0.484978

Ti -3.182744 1.573393 -0.096952

Ti 0.790205 -1.872552 1.245352

Ti 2.802077 -0.634741 -0.417555

Ti 0.201081 -0.221440 -1.561377

Ti -0.332252 2.744401 -0.344756

Ti -1.428954 0.196416 1.641452

Ti -2.713794 -0.792364 -1.654214

Ti 1.864663 1.775030 1.028508

O -2.350805 -1.514896 1.532137

O 3.414162 0.917822 0.104244

O -0.292634 -3.365816 1.487021

O 1.855155 -0.736787 -2.040263

O 2.299670 1.950566 2.558601

O -2.262010 3.013943 -0.511446

O -1.262503 -0.664844 -2.680531

O -2.988650 1.213335 1.681193

O 2.557548 -2.043018 0.783466

O -3.987004 0.535136 -1.399547

O -0.036565 -0.716411 2.426184

O -0.301744 1.720876 1.183626

O -1.784438 0.257744 -0.307436

O 1.143704 -0.069665 0.254296

O 0.153556 1.595197 -1.658199

O -0.313992 -1.849856 -0.377217

O 1.208234 3.418479 0.113922

O -2.884930 -2.442403 -1.010004

O 4.885279 -1.153902 -1.192526

C 5.824531 -0.467128 -1.096694

O 6.762121 0.178568 -1.019949

**(TiO2)10CO2 - (Ti9) – OPT- M06/DEF2SV(P)**


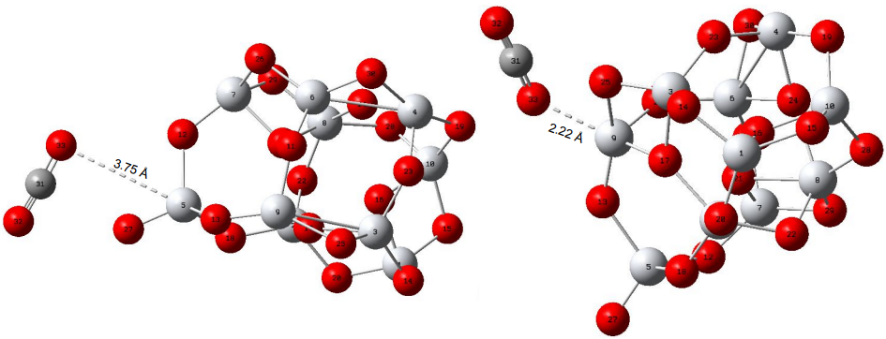


Ti -2.309399 1.013605 -2.371591

Ti 0.054337 -0.786511 -1.870697

Ti -0.478085 2.285716 -0.476535

Ti -1.964053 0.950376 2.270136

Ti 3.148928 -1.577633 -1.257002

Ti 0.492681 -0.310919 2.297439

Ti 1.292727 -2.801113 1.169335

Ti -1.339892 -2.202703 0.286502

Ti 2.193662 1.437315 -0.015790

Ti -3.514837 -0.165449 -0.103112

O 1.702606 0.885459 1.602098

O 2.694930 -2.869167 0.140160

O 3.315723 0.281931 -0.640923

O -1.595110 2.619225 -1.913008

O -3.976432 0.489832 -1.767898

O -1.756946 0.328566 -0.700097

O 0.695115 0.882599 -1.026372

O 1.578469 -1.385859 -2.389329

O -3.645253 0.701996 1.489769

O -1.066174 0.018332 -3.168566

O 0.516611 -1.300717 0.376326

O -0.977331 -2.260096 -1.442361

O -1.258176 2.173025 1.223550

O -1.363905 -0.692253 1.684269

O 1.018164 3.135830 -0.226234

O 1.330378 -1.836802 2.751259

O 4.438335 -2.035719 -2.078090

O -3.214077 -1.885391 0.143117

O -0.399549 -3.574588 0.991869

O -0.529694 0.690524 3.447819

C 3.303841 4.211369 0.613452

O 3.068386 5.320869 0.768386

O 3.611862 3.086493 0.470287
